# Viral meningitis epidemics and a single, recent, recombinant and anthroponotic origin of swine vesicular disease virus

**DOI:** 10.1093/emph/eov026

**Published:** 2015-10-27

**Authors:** Christian A. W. Bruhn, Sandra C. Abel Nielsen, Jose Alfredo Samaniego, Jemma Wadsworth, Nick J. Knowles, M. Thomas P. Gilbert

**Affiliations:** ^1^Centre for GeoGenetics, Natural History Museum of Denmark, Soelvgade 83 S, Copenhagen K, DK-1307, Denmark;; ^2^The Pirbright Institute, Ash Road, Pirbright, Woking, Surrey, GU24 0NF, UK;; ^3^Present address: Weinberger Lab, Laboratory of Epidemiology and Public Health, Yale School of Medicine, 60 College Street, 06510 New Haven, CT and; ^4^Stanford University, School of Medicine, 300 Pasteur Drive, CA 94305, USA

**Keywords:** emerging diseases, RNA viruses, *Enterovirus B*, viral meningitis, SVDV, *Picornaviridae*

## Abstract

**Background and objectives:** Swine vesicular disease virus (SVDV) is a close relative of the human *Enterovirus B* serotype, coxsackievirus B5. As the etiological agent of a significant emergent veterinary disease, several studies have attempted to explain its origin. However, several key questions remain, including the full biological ancestry of the virus, and its geographical and temporal origin.

**Methodology:** We sequenced near-complete genomes of 27 SVDV and 13 coxsackievirus B5 samples, all originally isolated between 1966 and 2006, and analysed these in conjunction with existing sequences and historical information.

**Results:** While analyses incorporating 24 additional near-complete SVDV genomic sequences indicate clear signs of within-SVDV recombination, all 51 SVDV isolates remain monophyletic. This supports a hypothesis of a single anthroponotic transfer origin. Analysis of individual coding and non-coding regions supports that SVDV has a recombinant origin between coxsackievirus B5 and another *Enterovirus B* serotype, most likely coxsackievirus A9. Extensive Bayesian sequence-based analysis of the time of the most recent common ancestor of all analysed sequences places this within a few years around 1961. Epidemiological evidence points to China as an origin, but there are no available samples to test this conclusively.

**Conclusions and implications:** Historical investigation and the clinical aspects of the involved *Enterovirus B* serotypes, makes the current results consistent with a hypothesis stating that SVDV originated through co-infection, recombination, and a single anthroponotic event, during large viral meningitis epidemics around 1960/1961 involving the ancestral serotypes. The exact geographical origin of SVDV may remain untestable due to historical aspects.

## Abstract (precis)

Swine vesicular disease, an important viral disease affecting domestic pigs, is shown to have a single and recent origin in humans, leading us closer to a full understanding of the sudden emergence of this enigmatic veterinary disease, and exemplifying the sometimes overlooked risk of human to animal disease transfers.

## INTRODUCTION

Swine vesicular disease virus (SVDV) is the etiological agent of swine vesicular disease (SVD) [1,2]. SVDV was initially isolated at two farms in Lombardy, Italy, in October 1966, where due to similarities in the symptoms of the affected swine, an initial diagnosis of foot-and-mouth disease (FMD) was made [1]. However, laboratory testing failed to support this diagnosis, and ultimately lead to the discovery of SVDV, and confirmation that it was the agent of the disease [1,2]. SVDV is a single-stranded (non-segmented) positive-sense RNA virus [3], typically of around 7400 bases, and classified as a member of the *Enterovirus B* species (family *Picornaviridae*). Although a pathogen of swine, previous studies have noted close similarity in the capsid region to another *Enterovirus B* serotype, coxsackievirus B5 (CV-B5)—an observation that is notable due to its status as a human pathogen linked to a range of cardiovascular and neurological pathologies [4–7]. Given this tantalising link, and in particular the suggestion that SVDV may have originated as an anthroponotic transfer (i.e. human to swine), several studies have previously attempted to identify the geographical, temporal, and biological origin of SVDV [7,8]. In this regard, Hong Kong has been postulated as the origin of SVDV, given it was (i) the second location in which SVDV was detected (in 1970), (ii) data support SVDV endemism in Hong Kong in subsequent years (Supplementary Table S1, showing SVDV geographical occurrence) and (iii) several lines of evidence indicate that SVDV has been introduced to Europe from Asia in separate events after 1970 [8]. Time-calibrated phylogenetic analyses of sequence data from both structural [1D (VP1)] and non-structural [3BC (VPg-protease)] regions of the genome, indicate that SVDV is monophyletic with respect to other serotypes, and had a last common ancestor between 1945 and 1965 [8]. Whether this date represents a time-window for the original anthroponotic transfer that lead to the establishment of SVDV in swine, or a severe bottleneck in a longer history of the virus is uncertain [8].

Complications exist, however, with the results of the previous studies. It has previously been discussed that SVDV and CV-B5 are either homologous across the genome, or only homologous in the capsid region (due to recombination), or that their similarity in the capsid region stems from convergent evolution [8]. *Enterovirus B* serotypes are notoriously recombinant viruses [9–11] and this fact has made it difficult to determine the ancestral lineage of SVDV outside the capsid region, regardless of whether one assumes recombination as a part of its origin or not. Because the external parts of the structural capsid region (P1 region: 1A (VP4) (internal), 1B (VP2) (external), 1C (VP3) (external), 1D (VP1) (external)) determine serology [4], analyses of this region will generally be expected to reveal taxa of the same serotypes as monophyletic [11,12]. However, this is not the case for the non-structural regions [P2 region: 2A, 2B, 2C. P3 region: 3A, 3B (VPg), 3C (protease), 3D (polymerase)], where monophyly of serotypes is typically only seen when the samples have a close spatiotemporal relationship [12]. This greatly complicates inference regarding the origin of SVDV—not only for the non-structural regions.

To address these challenges, we generated near-complete genome length sequences from a temporally distributed dataset of 27 SVDV and 13 CV-B5 isolates, and used this data to perform independent phylogenetic analyses on eight protein-coding regions and one non-coding region [the five prime untranslated region (5′UTR)] in order to assess both the biological and the geographical origin of SVDV across the full genome. Furthermore, we investigated the occurrence of within-SVDV recombination, and used the results to guide optimal Bayesian inference dating estimates of the most recent common ancestor of all existing full or near-full length sequenced SVDV strains. We subsequently placed these results in an *Enterovirus* epidemiological and historical setting in order to formulate a comprehensive, new and falsifiable hypothesis on the origin of SVDV. This integrated approach sheds further light on the dynamics involved when a pathogen emerges as the cause of a new disease via a cross species transfer, in this case an anthroponotic transfer to swine.

## METHODOLOGY

### Virus isolates selected

26 SVDV and 13 CV-B5 isolates grown in a pig kidney cell line (IB-RS-2) were selected for near-complete genome sequencing on the Illumina HiSeq platform, an additional SVDV isolate (HKN/19/70) was sequenced subsequently on the Illumina MiSeq platform, for a total of 27 SVDV isolates (Table 1 and Supplementary Table S4, for an overview of isolates included in this study). Isolates were chosen (within availability) to best reflect the known natural history of SVD. CV-B5 isolates were chosen with the goal of finding sequences closer related to SVDV, than those, which are already known. All isolates were obtained from The Pirbright Institute, UK, where they have been held as a result of past veterinary investigation into SVDV infected livestock.

**Table 1.** Samples sequenced in this study

**Sample no.**	**Virus**	**Isolate reference**	**Origin**	**Date collected**	**Accession no.**
1	SVDV	BUL/2/71	Plovdiv, Bulgaria	1971	KT284979
3	SVDV	GRE/1/79	Greece	July 1979	KT284980
6	SVDV	UKG/308/73	Farm H, Heaton Moor, Stockport, Gtr. Manchester, UK	30 October 1973	KT284981
8	SVDV	USS/6/72	Odessa region, Ukraine, USSR	1972	KT284982
10	SVDV	HKN/1/80	Tai Shui Hang, Lantau Island, Hong Kong	1 February 1979	KT284983
11	SVDV	HKN/7/81	Bing Kong, Sheung Shui, N.T., Hong Kong	6 January 1981	KT284984
12	SVDV	HKN/1/82	Shek Kong, Kam Tin, N.T., Hong Kong	2 April 1981	KT284985
13	SVDV	HKN/5/85	Ping Che, Ta Kwu Ling, Fanling, N.T., Hong Kong	28 December 1984	KT284986
14	SVDV	HKN/19/85	Mong Tseng Tsuen, Ping Shan, Y.L., N.T., Hong Kong	3 July 1985	KT284987
15	SVDV	HKN/12/87	Ma Tso Lung, Sheung Shui, N.T., Hong Kong	25 March 1987	KT284988
16	SVDV	MTA/22/75	Rabat, Zebbug, Malta	20 August 1975	KT284989
17	SVDV	ITL/A/89	Campodoro, Padova, Veneto, Italy	23 December 1988	KT284990
18	SVDV	ITL/1/91	Messina, Sicily, Italy	30.January 1991	KT284991
19	SVDV	ITL/2/91	Agerola, Naples, Campania, Italy	15 April 1991	KT284992
21	SVDV	ITL/16/2006	Offlaga, Brescia, Lombardy, Italy	1 December 2006	KT284993
22	SVDV	AUR/1/73	Wiener Neustadt, Austria	December 1972	KT284994
23	SVDV	POL/1/73	Yaslo District, Poland	13 December 1972	KT284995
24^a^	SVDV	HKN/19/70	Kwan Tei, Fanling, N.T., Hong Kong	9 March 1970	KT284996
25	SVDV	ITL/1/66	Lombardy, Italy	October 1966	KT284997
26	SVDV	HKN/36/71	Shui Tsan Tin, Pat Heung, Y.L., Hong Kong	29 April 1971	KT284998
28	SVDV	FRA/1/73	Bordeaux, France	January 1973	KT284999
29	SVDV	HKN/11/72	Ngau Tam Mei, Sun Tin, N.T., Hong Kong	29 February 1972	KT285000
31	SVDV	HKN/3/91	Fung Kut Heung, Kam Tin, Y.L., N.T., Hong Kong	6 July 1991	KT285001
32	SVDV	HKN/4/89	Ki Lun Shan, San Tin, Y.L., N.T., Hong Kong	19 March 1989	KT285002
33	SVDV	TAW/119/97	Kaoshiung, Taiwan POC	18 December 1997	KT285003
36	SVDV	ITL/3/73	Latina, Lazio, Italy	November 1972	KT285004
37	SVDV	ITL/5/77	Mantova, Lombardy, Italy	3 October 1977	KT285005
40	CV-B5	2137/70	Wisconsin, USA	1970	KT285006
41	CV-B5	4469/72	Georgia, USA	1972	KT285007
42	CV-B5	9030/77	Idaho, USA	1977	KT285008
43	CV-B5	4634/83	Alabama, USA	1983	KT285009
44	CV-B5	9954	Birmingham, UK	1973	KT285010
45	CV-B5	8068	Birmingham, UK	1973	KT285011
46	CV-B5	1603/Finland/82	Finland	1982	KT285012
48	CV-B5	93083-3/Taiwan/83	Taiwan	1983	KT285013
49	CV-B5	028/Pakistan/91	Pakistan	1991	KT285014
50	CV-B5	93-17428/France/93	France	1993	KT285015
51	CV-B5	84-6500/France/84	France	1984	KT285016
53	CV-B5	4267/Cambridge/92	Cambridge, UK	1992	KT285017
54	CV-B5	HONGKONG	Hong Kong	c. 1972	KT285018

a This sample was sequenced on the Illumina MiSeq platform, all other samples were sequenced on the Illumina HiSeq platform (see ‘Methodology’ section).

### RNA isolation, first-strand cDNA synthesis and PCR amplification

RNA was extracted using an RNeasy kit (Qiagen, Valencia, CA) followed by 1st strand cDNA synthesis using SuperScript III Reverse Transcriptase, RNaseOUT, and dNTPs (all Invitrogen, Carlsbad, CA). All components were mixed and briefly centrifuged before use. The following reagents were mixed in a 0.2 ml PCR tube at a total of 12 μl: 5 μl RNA, 2 μM oligo(dT) primer and 0.4 mM dNTPs. Samples were incubated 5 min at 65°C followed by a snap-chill on ice. To the RNA/primer/dNTP mix the following reagents were added to a total of 20 μl: 1× RT buffer, 10 mM DTT, 40 U RNaseOUT and 200 U SuperScript III RT enzyme. After a gentle mix and brief centrifugation samples were incubated 50 min at 50°C followed by 15 min at 70°C. To each sample, 1 μl RNase H was added, incubated for 20 min at 37°C, and transferred to ice. PCR amplification was performed as previously published [13].

### Sample fragmentation and preparation for export from the Pirbright laboratory

DNA concentration was quantified using a ND-1000 spectrophotometer (NanoDrop Technologies, Thermo Scientific, Wilmington, DE) prior to fragmentation using NEBNext dsDNA Fragmentase (New England Biolabs, Ipswich, MA). Fragmentation was performed as follows: 3 μg DNA from each sample was added to 1× Fragmentase Reaction buffer, 1× BSA, and nuclease-free water ad hoc to 54 μl. The reaction mix was subsequently vortexed thoroughly and incubated on ice for 5 min. Three units of dsDNA Fragmentase were added to the reaction and incubated 15 min at 37°C in order to generate fragments sizes of 600–800 bp. The incubation was stopped by addition of 5 µl 0.5 μM EDTA. Samples were purified using Qiagen’s PCR purification kit (Qiagen, Valencia, CA) according to manufacturer’s guidelines. Samples were eluted in 30 μl EB buffer. Correct fragment sizes were verified on the Agilent 2100 Bioanalyzer (Agilent Technologies, Santa Clara, CA) using a DNA7500 chip. Prior to sample export, 1/10 volume of sodium acetate (3M) was added as well as 2.5 volumes of absolute ethanol (calculated after addition of sodium acetate). Samples were incubated for 2 h at 56°C and washed with disinfectant (FAM 30®; Evans Vanodine International plc, Preston, UK).

### Ethanol precipitation

Amplicons from Pirbright containing sodium acetate and absolute ethanol were centrifuged at 14 000 g for 1 h at 4°C. Supernatant was carefully removed and discarded, leaving a DNA pellet. Pellets were dissolved and rinsed in 150 μl ice-cold ethanol (70%) and afterwards centrifuged again for 15 min. Supernatant was discarded and pellet was dried for 10 min at 65°C before being dissolved in 85 μl EB buffer.

### High-throughput deep sequencing

Samples were fragmented further to meet the desired insert size for sequencing on the Illumina platform, using the Bioruptor Sonication System (Diagenode, Denville, NJ) with the setting: High intensity, 30″/30″, 20 cycles. Resulting fragment sizes were analysed using a High Sensitivity chip on the Agilent 2100 Bioanalyzer.

Independent sequencing libraries were produced on each sample using New England Biolabs’ NEBNext DNA Sample Prep, Master Mix Set 2. Samples were subsequently pooled and sequenced on 2 lanes of an Illumina HiSeq2000 (100SR).

### Processing of raw data and sequence assembly

Using AdapterRemoval (at the time of application the program was called SinglEndPrimeRemoval3-5M) [14] raw sequence reads were trimmed if they contained N’s, had a Phred Sanger score <35, or if the adapter sequence aligned to the read using default settings. Reads shorter than 25 bps were also removed. The cleaned sequences were checked with FastQC [15] followed by removal of duplicates using PRINSEQ [16].

Processed reads were mapped using a workflow described previously [17], but adjusted to the high number of reads obtained from the Illumina HiSeq platform (unique read numbers ranged from 465 568–4 727 739 per isolate). *De novo* assembly was only used for an initial assessment of the workflow on the data set. Final consensus sequences were obtained by using three different iterative mappings for all isolates (except HKN/19/70, see below), the first with SVDV acc. X54521 isolate UKG/27/72 as the reference, using the standard ‘Medium Sensitivity’ setting in Geneious 6.0.3 [18] and allowing up to 25 iterations, the second mapping was against the same reference but using the standard ‘Low Sensitivity’ setting and allowing up to 100 iterations. Finally isolate reads were mapped with CV-B5 acc. X67706 isolate 1954/UK/85 as the reference, using the ‘Medium Sensitivity’ setting and up to 25 iterations (some of these mappings were allowed to run for up to 100 iterations, depending on mapping convergence). The consensus sequences were obtained using a strict 50% criterion for the base-calls. The final consensus sequence for each isolate was then obtained by aligning the consensus sequences for each of the three mappings and calling the bases using a 100% strict criterion (in a few cases, a mapping consensus was left out of this final alignment due to obvious error in the parent contig). Three of these samples (CV-B5/93083-3/Taiwan/83; CV-B5/028/Pakistan/91; CV-B5/93-17428/France/93) were additionally *de novo* assembled using DNAstar SeqMan NGen version 12 [19]. The processed fastq files were sampled using 200 000 reads. The following parameters were: GenomeLength: 7000; MaxGap: 6; MatchSize: 21; MatchSpacing: 50; and MinMatchPercent: 93. In each case a full-length contig was generated and exported as a fasta file. The relevant fasta file was then used as a template to examine the whole fastq dataset in a templated assembly using the same software. Depth of coverage was generally between 10 000 and 20 000. This was done to assess the occurrence of a few degenerate calls within these sequences using the former method (degeneracies were included in analyses). The isolate HKN/19/70 was sequenced on the Illumina MiSeq Platform (150 bases/paired end/Nextera XT) and *de novo* assembled using DNAstar SeqMan NGen version 11.2.1 [19].

Trimming sequences within the primers left them with a norm length of 6983 bp, including the entire coding region and a further 307 bases upstream and 73 bases downstream (excluding stop codon).

### Recombination analysis

Within-SVDV recombination analysis was performed by aligning the 27 SVDV sequences from this study with those from all 24 existing SVDV isolates having complete or near-complete genome length sequences available (Table 1 and Supplementary Table S4). Alignment was performed with the MAFFT v. 7.017 [20,21] plug-in in Geneious 6.0.3 [18] with subsequent visual inspection and minor manual editing, leaving the 51 sequence alignment 7412 bases long. This basic SVDV alignment (BSA) was then used to construct a set of derived alignments either non-randomly (by selection a genome section and extracting this) or pseudo-randomly (by reducing the number of sequences in the alignment), or in a few cases by a combination of the above, for a total of 15 alignments (Supplementary Table S2). All alignments were run in GARD [22,23] on the server provided by www.datamonkey.org [24] (29 December 2013, date last accessed), either to convergence or to the maximum server allowance cut-off. A single genome-wide plot was then constructed showing all recombination signals detected across all 15 analyses and their levels of support (Kishino-Hasegawa [25]) (Supplementary Fig. S1).

### Phylogenetic analysis of genomic sections

**Figure 1.**
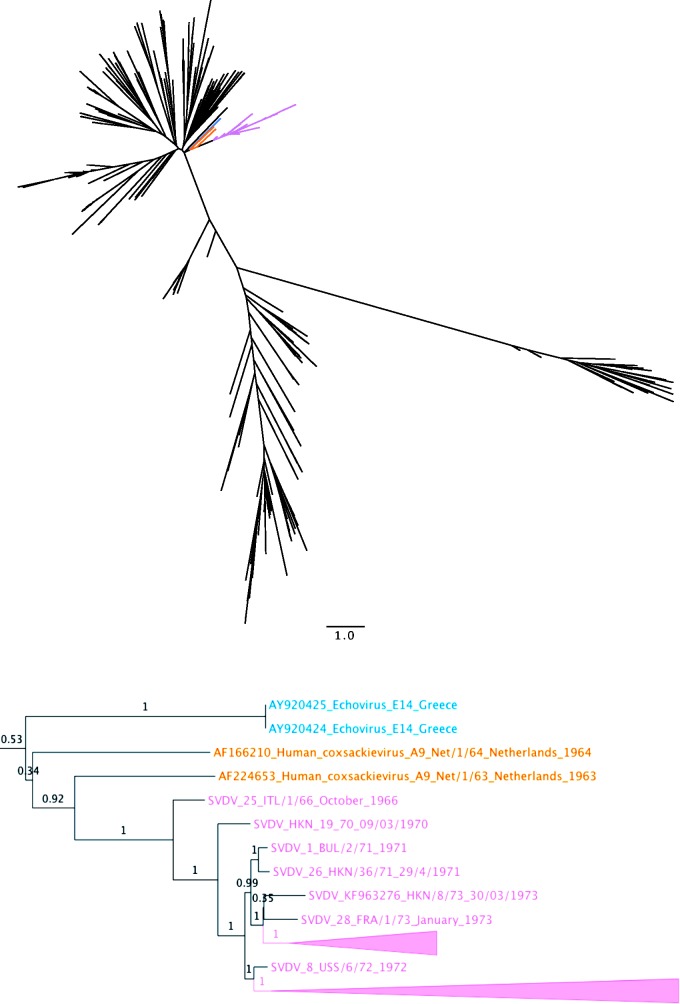
Maximum likelihood tree for the 3D (Polymerase) genomic section Result from the maximum likelihood phylogenetic analysis of the 3D (Polymerase) genomic section (see also Supplementary Table S5 and Methodology). Showing—on top—an unrooted tree with no taxa designations, except that branches leading to an SVDV isolate are coloured pink, and closest related samples are shown in orange (Dutch CV-A9 samples) and blue (Greek echovirus serotype E14 samples), giving an immediate overview of the relation between SVDV and all other sequences in the analysis, including relative (and if conferring with the 0.1 substitution-per-site bar, also absolute) distance to the nearest neighbours. Below, a cut-out of a mid-point rooted versions of the same tree. The earliest SVDV isolate (ITL/1/66) is seen as sister to all other SVDV isolates at the root of the monophyletic SVDV cluster. The early Dutch, 1963, CV-A9, isolate (Net/1/63 acc. AF224653) is seen as a strongly supported sister of all SVDV sequences (0.92 aBayes [35])

To determine the closest related (sequenced) virus strains to SVDV across the genome, the BSA was used to obtain 9 further derived alignments, corresponding exactly to the following genomic sections: 5′UTR, 1A (VP4), 1B (VP2), 1C (VP3), 1D (VP1), 2A, 2C, 3C (protease), 3D (polymerase). For each of these alignments all 51 taxa were compared against Genbank [26] entries using BLAST [27] under standard settings, except that sequences with taxon ID: 12075 [SVDV] were excluded, and that for each alignment the number of sequences retained from each query was adjusted to either 10, 50 or 100 depending on an *ad hoc* assessment of the number of closely related sequences in the database for that particular region. This was done to exclude very distant sequences from the analysis. For each of the nine sections, BLAST [27] results from all 51 taxa were downloaded, duplicates with respect to accession number were removed, but duplicate sequences were left (to avoid inadvertent removal of entries with superior annotations), short matches were also removed, with a cut off of 150, 200 or 250 bases depending on the section. The BLAST [27] hits were aligned with the queries and with the corresponding sequence section from the 13 CV-B5 isolates sequenced in this study (and 3 further CV-B5 isolates previously sequenced but suspected of contamination). Alignment was performed using the same methodology as for the recombination analysis, (Supplementary Table S5). All alignments were tested in jModelTest v. 2.1.1 [28,29] allowing 4 gamma categories, invariant sites and unequal frequencies under 11 different substitution schemes. They were assessed under the Akaike [30], corrected Akaike [31] and Bayesian information [32] criteria (AIC, AICc and BIC) as well as the decision theoretic performance-based selection criterion (DT) [33]. For situations yielding differing results across criteria the BIC and DT were adhered to. BIC and DT were in accord across all analyses (Supplementary Table S5). The chosen model (for each alignment) was implemented for maximum likelihood based phylogenetic tree construction in GARLI v 2.0 [34] using three search replicates. Branch supports (aBayes [35]) were obtained in PhyML v. 20110526 [36] by fixing the topology, and parameters obtained from the best search replicate in GARLI, but with allowance for branch length optimization; except for the 3C alignment where a separate bootstrap run (100) was performed in GARLI and annotated onto the best search replicate using TreeAnnotator v. 1.8.0 [37]. All 9 trees were visualized using FigTree v. 1.4.0 [38] (Fig. 1 and Supplementary Fig. S2).

### Bayesian dating estimates

Timing of events in the SVDV phylogeny was estimated using a Bayesian statistical approach, as implemented in the BEAST v.1.8.0 package [37]. The 51 taxa full length BSA was assigned tip dates according to the time of sample collection. Isolates lacking a precise date were coded with the ‘set precision’ function in BEAUti v. 1.8.0. [37] (e.g. when the date was given as a month in a year, the first of that month was entered as date with a forward ‘precision’ of 0.083). Two alignments extracted from this dated BSA were then constructed, corresponding to two different sections of the genome based on the results from the recombination analysis (Supplementary Fig. S1 and Supplementary Table S2). One from within the 1C (VP3) to 1D (VP1) section (400 full codons, positions 1881–3080 in SVDV H/3’76 D00435) and the other spanning the entire 3C (protease) to 3D (polymerase) region (645 full codons). These were analysed under five different tree priors (4 coalescent [39–42], and an epidemiological birth-death prior [43]) and using 3 different molecular clocks [44,45]. For a total of 30 analyses (Table 2)). All analyses were run under the SRD06 model of sequence evolution [46]. All analyses ran for 40 000 000 steps, sampling every 4000 [40]. All parameters for all analyses converged, and the minimum ESS value for any parameter in any run was > 200, as assessed by using Tracer v.1.5 [47] with a burn-in of 10%. Path Sampling (PS) [48,49] and Stepping-Stone (SS) [50] analyses were performed for all analyses to establish marginal likelihoods [51], using 64 steps and a chain length of 1 000 000, sampling every 100 (Table 2). Trees for the analyses with the highest marginal likelihood for each section and for both coalescent and epidemiological birth-death tree priors (for a total of 4, (in bold, Table 2)) were summarized in TreeAnnotator v.1.8.0 [37], using the maximum clade credibility criteria, a posterior probability limit of zero, median heights and a burn-in of 10%, and visualized in FigTree v.1.4.0 [38] (Supplementary Fig. S3). Priors used in the analyses can be found in Supplementary Table S6.

**Table 2.** Bayesian dating estimates

	Alignment	Partial capsid alignment. 400 codons within 1C-1D (VP3-VP1). SRD06 codon partition model for nucleotide substitution		Full Protease 3C—Polymerase 3D alignment. 645 codons. SRD06 codon partition model for nucleotide substitution	
**Tree prior**	***Molecular Clock***	**Marginal likelihood** [log(10) values]	**Age of root** [Decimal calendar years]	**Marginal likelihood** [log(10) values]	**Age of root** [Decimal calendar years]
		SS	Mean/Median (95% HPD)	**SS**	Mean/Median (95% HPD)
		PS		PS	
		SS–PS		SS–PS	
		SS-best–SS-current		SS-best–SS-current	
**Coalescent Constant Size**	*Strict*	−6175.92769	1961.55/1961.50	−9239.50417	1959.22/1959.27
		−6176.26690	(1959.73–1963.23)	−9239.93468	(1957.16–1961.06)
		0.3392		0.4305	
		57.1		78.0	
	*Uncorrelated Lognormal Relaxed*	−6124.19152	1957.80/1958.58	−9166.40662	1954.50/1955.53
		−6124.49187	(1949.90–1963.81)	−9166.66545	(1943.79–1962.64)
		0.3002		0.2588	
		5.33		4.94	
	*Uncorrelated Exponential Relaxed*	−6125.13136	1955.57/1957.32	−9163.73276	1952.42/1954.68
		−6125.56094	(1942.13–1964.72)	−9164.14054	(1935.18–1963.58)
		0.4296		0.4078	
		6.27		2.27	
**Coalescent Exponential Growth**	*Strict*	−6173.90996	1961.48/1961.53	−9240.11241	1959.22/1959.27
		−6174.26098	(1959.74–1963.26)	−9240.88384	(1957.29–1961.15)
		0.3510		0.7714	
		55.1		78.6	
	*Uncorrelated Lognormal Relaxed*	−6124.85464	1957.50/1958.23	−9164.29592	1954.60/1955.63
		−6125.06475	(1949.74–1963.92)	−9164.84035	(1944.27–1962.99)
		0.2101		0.5444	
		6.00		2.83	
	*Uncorrelated Exponential Relaxed*	−6124.24950	1953.77/1956.00	−9164.85397	1951.49/1953.92
		−6124.32176	(1937.57-1964.22)	−9165.35887	(1934.12–1963.94)
		0.0722		0.5049	
		5.39		3.39	
**Coalescent Logistic Growth**	*Strict*	−6178.50005	1961.53/1961.60	−9240.90492	1959.26/1959.29
		−6178.82343	(1959.67–1963.18)	−9241.40787	(1957.29–1961.21)
		0.3234		0.5030	
		59.6		79.4	
	*Uncorrelated Lognormal Relaxed*	−6126.80959	1957.89/1958.67	−9167.96309	1954.19/1955.30
		−6126.91267	(1949.95–1964.05)	−9168.60143	(1943.54–1962.95)
		0.1031		0.6383	
		7.95		6.50	
	*Uncorrelated Exponential Relaxed*	−6127.11748	1955.48/1957.15	−9166.95847	1953.06/1954.82
		−6127.66222	(1941.97–1964.45)	−9166.96760	(1938.69–1963.67)
		0.5447		0.00913	
		8.26		5.49	
**Coalescent Bayesian Skyline**	*Strict*	−6167.89123	1961.85/1961.91	−9228.88172	1959.47/1959.52
		−6168.16191	(1960.05–1963.48)	−9229.35113	(1957.55–1961.37)
		0.2707		0.4694	
		49.0		67.4	
	*Uncorrelated Lognormal Relaxed*	**−6118.85855**	**1960.25/1960.84**	**−9161.46459**	**1957.44/1958.14**
		**−6119.00140**	**(1954.35–1965.23)**	**−9161.45594**	**(1949.51–1964.11)**
		**0.1429**		**−0.0087**	
		**0.00**		**0.00**	
	*Uncorrelated Exponential Relaxed*	−6121.10564	1958.81/1960.49	−9162.02247	1957.90/1959.32
		−6121.26521	(1947.82–1965.90)	−9162.18815	(1947.02–1965.23)
		0.1596		0.1657	
		2.25		0.56	
**Epidemiology Birth-Death Basic Reproductive Number**	*Strict*	−6169.06252	1962.06/1962.11	−9233.63644	1959.80/1959.85
		−6169.64219	(1960.47−1963.67)	−9234.08112	(1957.96–1961.56)
		0.5797		0.4447	
		50.2		72.2	
	*Uncorrelated Lognormal Relaxed*	**−6120.52255**	**1962.65/1962.85**	−9163.50126	1961.42/1961.65
		**−6120.90851**	**(1959.68–1965.39)**	−9164.01821	(1957.89–1964.73)
		**0.3860**		0.5170	
		**1.66**		2.04	
	*Uncorrelated Exponential Relaxed*	−6121.87552	1963.02/1963.26	**−9163.38768**	**1962.38/1962.63**
		−6122.19743	(1959.90–1965.66)	**−9163.73844**	**(1959.06–1965.27)**
		0.3219		**0.3508**	
		3.02		**1.92**	

Timing of events in the SVDV phylogeny estimated using a Bayesian statistical approach. The 51 taxa full-length basic SVDV alignment (BSA) was assigned tip dates according to the time of sample collection. Two alignments extracted from this dated BSA were then constructed, corresponding to two different sections of the genome based on the results from the recombination analysis—one from within the 1C (VP3) to 1D(VP1) section (400 full codons, positions 1881–3080 in SVDV H/3′76 D00435) and the other spanning the entire 3C (protease) to 3D (polymerase) region (645 full codons). These were analysed under five different tree priors using three different molecular clocks. All analyses were run under the SRD06 model of sequence evolution [46]. PS [48,49] and SS [50] analyses were performed for all analyses to establish marginal likelihoods [51]. Highest marginal likelihood results for each section and for both coalescent and epidemiological birth-death tree priors are shown in bold (see also Supplementary Fig. S3 and Methodology).

HPD, Highest posterior density interval.

## RESULTS

### Recombination analysis

Within-SVDV recombination analysis was conducted to ascertain which genomic regions were free of recombination signals, and thus suitable for use in the Bayesian dating estimates analyses. Of the 15 recombination analyses performed to ensure increased detection sensitivity and for lowering the risk of genomic regions being falsely negative for recombination signals (Supplementary Table S2 and Supplementary Fig. S1), all except numbers 7 and 8 (which are both limited to genome region P3 and the 3′UTR) yielded signals of recombination. Recombination signals were distributed non-randomly across analyses and the genome. Several recombination signals did not pass the Kishino-Hasegawa [25] test for significance. This included one located at the transition between the highly conserved internal ribosome entry site and the hyper-variable spacer region [52] in the 5′UTR (genome position approximately 570, SVDV H/376 D00435), and a second at approximately position 818, which is roughly one third into the short 1A (VP4) protein coding region. Highly supported signals are found at both the beginning and the end of the 1B (VP2) protein coding region; towards the last quarter of 1D (VP1); within 2A; possibly within the middle of 2C; and finally in 3A. The alignment is free of recombination signals downstream of just prior to the start of 3C. This combined information was used to select two genomic sections free of recombination signals for subsequent dating analysis, one from within the 1C (VP3) to 1D (VP1) section (400 full codons, positions 1881–3080 in SVDV H/3′76 D00435) and the other spanning the entire 3C (protease) to 3D (polymerase) region (645 full codons).

### Phylogenetic reconstruction of genomic sections

SVDV sequences were monophyletic in eight of the nine genomic subsections analysed (Fig. 1 and Supplementary Fig. S2). The final analysis, 1A (VP4), is based on the shortest alignment (207 bases), and it is noteworthy that some of the sequences which break the monophyly are also among those found to be closest to SVDV in the other capsid region analyses. For the 5′UTR there is a long branch leading to the SVDV cluster, and there are no sequences distinctly closest to the cluster. The sequences in that particular analysis are distinguished by being from a very diverse set of *Enterovirus B* viruses, but with only very few CV-B5 isolates (relative to capsid region analyses).

Overall, the largest sequence dataset that we could compare our results to was the one for genomic region 1D (VP1). In this regard, the phylogenetic association recovered is in agreement with that published by Gullberg et al. (2010) [53], and the SVDV cluster falls within the CV-B5 subgenogroup A1 following the nomenclature of Henquell et al. (2013) [54]. Within this cluster we also placed CV-B5 sequences from Taiwan, Japan and Belarus, in addition to the geographically diverse cluster of sequences from [South] Korea, Romania, Germany, Netherlands, France, Denmark and China that were previously placed there by Henquell et al. (2013) [54]. Generally, for all three external capsid regions [1B (VP2), 1C (VP3) and 1D (VP1)], we find that the monophyletic SVDV cluster is nested within highly supported purely CV-B5 clusters, whereas this is not the case for the internal capsid region 1A (VP4). As the internal capsid region is very short - and thus not highly phylogenetically informative - the importance of the associations of SVDV, including with CV-B5, CV-A9, CV-B4 and echovirus serotype E30, found here is difficult to assess. Of some note, is the position of the newly sequenced Taiwanese 1983 CV-B5 isolate, 93083-3/Taiwan/83, which is closely related to SVDV in all four capsid regions and also shows up as closely related to SVDV in the 2A region. However, downstream of the capsid the results of the analyses change dramatically. Specifically, the SVDV cluster is no longer nested within CV-B5 clusters, as was the case for the 3 external capsid regions. For region 2A, the SVDV cluster falls within a highly supported cluster with *Enterovirus B* species sequences from echovirus serotypes E6, E9, E25 and E30, as well as CV-A9, and as mentioned, the 93083-3/Taiwan/83 CV-B5 isolate. However, the branch leading to the SVDV cluster is quite long (relative to within SVDV distances) in this analysis. This latter observation also holds to some extent true for the 2C region, where the closest sequence is an echovirus 6 from Romania, with currently unknown year of isolation. For the 3C region the closest sequences to SVDV fall in an unsupported cluster (N.B. this is the only analysis which used bootstrap for support) including echovirus serotypes E9, E11 and E30 and once again with a single CV-B5 isolate from this study, this time a French 1984 isolate (84-6500/France/84). Finally the 3D polymerase tree shows the only result, where the closest (and highly supported sister) sequence to the SVDV cluster predates the 1966 discovery of SVD (Fig. 1). This 1963 Dutch CV-A9 isolate, Net/1/63 (acc. AF224653), stems from a patient suffering from fever and convulsions [55]. The sequence is 147 codons long and differs from SVDV ITL/1/66 by two amino acids (one being the initial codon of the sequence) and has a (patristic) nucleotide similarity of 92.1% (amino acid 98.6%). Another Dutch sample from the subsequent year of 1964, isolate Net/1/64 (acc. AF166210) from a patient suffering from gastroenteritis and pharyngitis [55], falls as an unsupported sister to SVDV and Net/1/63 (Fig. 1).

Taken together these results support a hypothesis, stating that SVDV stems from a single recombinant origin involving two *Enterovirus B* serotypes, CV-B5 and most likely CV-A9. Furthermore, SVDV has remained monophyletic across the genome, i.e. there is no supporting evidence of further recombination with *Enterovirus B* serotypes, or any other strains outside SVDV, following the emergence of SVDV in swine.

### Bayesian dating estimates

Thirty different Bayesian analyses were performed, exploring five different tree priors and three different molecular clocks on two separate genomic sections, both free of recombination signals (Table 2). Annotated trees from the highest marginal likelihood analyses for each of the two analysed genome sections and for both coalescent [39,40] and epidemiology birth-death tree prior analyses [43] were constructed (Supplementary Fig. S3, corresponding analyses in bold in Table 2). The difference in marginal likelihood in log(10) values between SS [50,51] and PS [48,49,51] ranges from −0.0087 to 0.7714 across analyses (Table 2). With SS being the faster converging process of the two, we take this to indicate, that the SS results can be used as a reliable measure for marginal likelihood due to convergence [56]. The difference in (SS) log(10) marginal likelihood between the model with the highest marginal likelihood and all other models (for the same genomic section) is equivalent to a Bayes factor [51,57,58] with the result given in the unit of bel. From this, it is clear that all analyses using a strict clock can be rejected outright—this is also supported by analysis of the ‘coefficient of variation’ parameter histograms in Tracer v. 1.5 [47]. Within the coalescent analyses, the Bayesian skyline with an uncorrelated lognormal relaxed clock, can reject all other analysed (non-skyline) tree priors with a factor of at least 2.27 bel for the protease-polymerase analyses and at least 5.33 bel for the within-capsid alignment. The best scoring epidemiology birth-death models use an uncorrelated log-normal clock for the within-capsid alignment and the relaxed uncorrelated exponential clock for the protease-polymerase alignment; these are both within 2 bel of the best coalescent model (Bayesian skyline, uncorrelated lognormal relaxed clock). Keeping to the best coalescent skyline [39,40,42] and epidemiological birth-death models [43] (in bold, Table 2), it is clear from the dates, that there is a very good correspondence between the estimates obtained from the two different genomic sections. The 95% highest posterior density interval (HPD) is much shorter for the epidemiology birth-death analyses with the oldest end not older than the beginning of 1959 for the best model for either of the genomic sections. Interestingly all four analyses support a scenario where the date of the most recent common ancestor of all analysed SVDV falls very close to the first isolation of SVDV (Table 2 and Supplementary Figs. S3 and S4). The range of the medians for the four analyses is from February 1958 to November 1962, and the combined range of their 95% HPDs on the age, is from mid-1949 to May 1965.

## DISCUSSION

### A recombinant origin for an intra-specifically recombining SVDV

Recombination between *Enterovirus B* species serotypes has previously been well documented and results in a loss of monophyly (with regard to serotype) outside of the capsid region [9,10,12]. Despite this, we found no evidence for SVDV recombination with other sampled viruses - although intra-specific SVDV recombination was apparent. We hypothesise this pan-genome SVDV monophyly may have been assisted by the host species barrier between SVDV and the *Enterovirus B* serotypes, with which the ancestral strains of SVDV would be expected to recombine. Obviously this same species barrier has been breached at one point—the origin of SVDV. It has long been suspected that this involved a recombination event between CV-B5 and one (or more) other unspecified *Enterovirus B* serotypes [7,8] (potentially even another *Enterovirus* species). Because of these very recombination dynamics, it is difficult to ascertain which polymerase sequence was associated with which serotype when going 50 years back in time—except if one is fortunate enough to have old sequenced samples. In this regard, the Dutch 1963 Net/1/63 isolate [55] is a remarkable find, both because of its close similarity to (the oldest) SVDV, and because it uniquely predates the first isolation of SVDV with an age that falls perfectly within the uncertainty intervals of our best-model dating analyses. Due to the spatiotemporal clustering of sequences (even) outside the capsid, this clearly suggests that SVDV arose as a recombinant between two *Enterovirus B* serotypes, with the first being CV-B5 and the second serotype most likely being CV-A9. A recombination origin is further supported by the overall contrast between the non-structural versus structural section results in our maximum likelihood tree analyses. The study [55], which generated the partial polymerase sequence from Net/1/63 did not analyse any other section than this downstream of section 2A, thus we do not know what the relations to SVDV are in the remainder of the 3rd genome region (i.e. it explains why we do not see this strain in the 3C analysis, assuming that recombination occurred upstream of this).

### Early isolations of SVDV in relation to the true geographical origin

If the above findings are taken as a starting point for a hypothesis for the origin of SVDV, then several questions remain to be answered. If the ancestral SVDV strain in pigs arose even a few years before the first isolation, how did it go unnoticed until October 1966? At SVDV’s original isolation on two farms in Lombardy, Italy, the disease was taken to be FMD due to clinical features being highly similar between the two [1]. It was only upon failure to detect FMD virus from the samples that further investigations were conducted, resulting in Nardelli *et al.’s* [1] correct diagnosis of an enteroviral agent in their September 1968 publication. Remaining ‘undercover’ as the agent of one of the most notorious veterinary diseases might not intuitively seem like an obvious way for a virus to have avoided discovery, but it clearly depends on the prevalence of the disease with which symptoms are shared, and also the level of surveillance and sampling. As an intriguing example, it can be seen (Supplementary Table S3) that at around the time of the Netherlands 1963 CV-A9 Net/1/63 isolate, i.e. from about 1962 and continuing until 1966, outbreaks of FMD were in the thousands in the country, whereas they were orders of magnitude lower only a few years before and after. This period fits not only with the age of the sample but also our dating of the SVDV ancestor. Under such conditions it is possible to imagine how an unknown virus with highly similar clinical manifestation could be overlooked. Other factors affecting such a scenario include which cell lines were being used for passage in monitoring labs, exemplified by FMDV being able to replicate in primary bovine thyroid cells, which is not the case for SVDV. However, any vesicular disease occurring in farm animals in Western Europe would be expected to be investigated, and SVDV would thus not be expected to go undetected for long, except possibly in the high incidence FMD settings mentioned earlier. Therefore it is reasonable to consider whether the more parsimonious explanation is simply that the origin lies in an area where there was no monitoring, or from where the results of monitoring have not been reported. The most obvious candidate for this is China. There is a complete lack of concurrent veterinary information or samples from China, and yet pig export patterns fit with several distinct introductions/occurrences of SVDV in Europe, Hong Kong and other parts of Asia. For Europe this may fit with the original isolated cases at the two farms in Italy, but more clearly so with later cases in eastern European countries, which were importing pig meat from China during the Cold War. Examples of this include SVDV in 1971 from a military farm in Plovdiv Bulgaria, where the origin was reported as pig meat from China, and where the BUL/1/71 strain is closely related to a Hong Kong strain from the same year (HKN/36/71). These strains are also related to samples from Odessa, Ukraine from 1972. Eastern Europe would then have served as a stepping stone for introductions into Western Europe as seems to have been the case in 1972, where classic epidemiology points to an introduction into Poland spreading into Austria and from there on to Italy, UK and eastern France. At the same time we recognize Hong Kong, where several of the oldest SVDV samples are from, as a major importer of pigs from China, as well as China being a likely origin for the 1973 introduction of SVDV into Japan. Thus, China needs to be considered as a strong candidate for the origin of SVDV.

### The biological and the geographical origin of SVDV

We also need to answer which events could have facilitated this recombination event, and where. There is no strong evidence to support whether recombination took place in humans or in pigs, but given that the ancestral strains are hosted by humans; it is the most parsimonious explanation that the recombination also took place in a human. Regardless of this, the most likely origin is a location with a coincident high prevalence of CV-B5 and CV-A9, at the appropriate time, and obviously where an appreciable level of swine farming was taking place—areas with early SVDV cases being the primary candidates. The distinct phylodynamics of *Enterovirus B* serotypes are helpful in narrowing this down. As already underlined in 1958 in the following quote by Albert Sabin [59]:

‘Please remember that Coxsackie B5 represents only one of more than 50 known viruses that may compete for the intestinal tract of children and their parents during the summer period. It is one of the interesting epidemiological manifestations that in one particular area one of these viruses can get the inside track and somehow push most of the others off.’

A particularly well-described example of this effect of acquired immunity, involving CV-B5 and CV-A9, is documented in Japan in 1960 and 1961 in connection with large outbreaks of aseptic (viral) meningitis [60–62], which is a serious, and unfortunately not uncommon clinical manifestation of several *Enterovirus B* serotypes. The particularly interesting thing about the 1960/1961 Japanese outbreaks in the present context (in addition to the fact that Japan is an early SVDV incidence country, although outbreaks of disease were limited to November–December 1973 and 1975) is that it is known that no other major epidemic of CV-B5 occurred in Japan until 1984 [63] and also, that no other CV-B5 epidemic had occurred for several years before [60–62]. This places the Japanese 1960/1961 CV-B5 and CV-A9 outbreak in a ‘time-bubble’ in perfect accord with the estimated origin of SVDV. Interestingly, Japan is not the only early-incidence SVDV location having CV-B5 and CV-A9 outbreaks documented in 1960/1961. Hong Kong, where SVDV was found in 1970 (Supplementary Table S1) as the earliest location subsequent to the initial isolations in Italy, also has a report of outbreaks of aseptic (viral) meningitis-causing CV-B5 (generally 1960) and CV-A9 (generally 1961) amongst British Service personnel, during the same years [64]. However, this particular report does not cover the general Hong Kong population (leaving it up to the reader to extrapolate to the true prevalence), also there is overall a one season time separation between the two strains in this report [64]. The alluring aspect of large aseptic meningitis outbreaks as a facilitating factor lies not only in the common biology with SVDV, but also in the simple fact that the higher the prevalence of the involved strains, the higher the risk of co-infection, recombination and subsequent anthroponotic emergence in swine will have been. This brings us to the fact, that of the three main aspects of the origin of SVDV, the biological, the geographical and the temporal—the geographical remains the most difficult to answer. Previous studies have pointed the finger in the direction of Hong Kong as the most likely location for an origin [8]. Our analyses (not shown) are in agreement with prior results showing that SVDV has been imported to Europe from Asia (from the 1970s and forward) [8], subsequent to the initial isolation in Italy in 1966, as mentioned above. However, we do not find that an origin in Europe can be categorically ruled out. The finding of the Dutch Net/1/63 strain as the closest sequence to SVDV in the polymerase region illustrates this, even if it seems to be negated by the almost vanishing historical incidence of SVDV in the Netherlands (also in later FMD-free years (Supplementary Tables S1 and S3)). The original Nardelli article [1] states that the two 1966 affected Italian farms ‘had received pigs for fattening from a common source’. The elusive nature of this ‘common source’ in the literature is quite frustrating, but the fact that they are received ‘for fattening’ could imply that it is not a very distant supplier. Strictly speaking, the case is still that we only know that SVDV has an origin in East Asia, most likely China, or potentially in Europe. The best way to specify this further (besides successful tracking of the quote from the Nardelli article [1]), would be to obtain historical samples from the type of outbreaks and period described earlier, sequence them, and perform phylogenetic analyses similar to the ones found in this study.

## CONCLUSIONS AND IMPLICATIONS

It needs to be reiterated, that the recombinant nature of the *Enterovirus B* serotypes strongly affects phylogenetic inference between serotypes for genomic sections outside those, which determine the serotype itself. This means that the further isolates are separated in time and space, the more likely they are to be non-monophyletic in non-structural regions with regards to their serotype designation. Thus, it cannot be outright rejected, that the Net/1/63 CV-A9 isolate has a genome section, which a few years previously was circulating in a (SVDV related) CV-B5 (or other serotype) strain. What we do know is that the sequence was fit in a CV-A9 serotype in 1963 and that a very closely related sequence was fit in a SVDV serotype in 1966. Given this, we conclude that a CV-B5 and CV-A9 recombination event leading up to the emergence of SVDV is a parsimonious explanation for the SVDVs ancestry. It is not clear if the recombination took place in humans or pigs, but with humans as the natural host and reservoir for the ancestral strains, humans are also the most likely mixing vessel.

Our extensive sequence based analyses indicate that SVDV arose within a narrow time-span around 1961. This result lends itself particularly well to suggesting large coinciding outbreaks of viral meningitis, caused by the ancestral serotypes, as documented in the most likely geographical origin of East Asia, as the historically contingent background for the emergence of this significant veterinary disease.

These results not only have significant implications for the understanding of the natural history of SVDV, but also represent an extraordinary example of a founding event, and fundamental change of ecology for this *Enterovirus B* serotype variant. The genome-wide monophyly of SVDV with regards to its closest relations, and across several decades, is extraordinary within this highly recombinant species. This means that the data and analyses given here might well prove to describe the birth of a new species.

## SUPPLEMENTARY DATA

Supplementary data is available at *EMPH* online.

Supplementary Data
