# Supplementary material for: Viral meningitis epidemics and a single, recent, recombinant and anthroponotic origin of swine vesicular disease virus
Source: Evol Med Public Health. 2015 Oct 27;2015(1):289–303. doi: 10.1093/emph/eov026 (PMC4661520; doi:10.1093/emph/eov026)
Supplement: Supplementary Data [file supp_2015_1_289__index.html]

Viral meningitis epidemics and a single, recent, recombinant and anthroponotic origin of swine vesicular disease virus — Supplementary Data 

# Viral meningitis epidemics and a single, recent, recombinant and anthroponotic origin of swine vesicular disease virus

## Supplementary Data

files

- Supplementary Data - pdf file
- Supplementary Data - pdf file
