# Supplementary material for: Viral meningitis epidemics and a single, recent, recombinant and anthroponotic origin of swine vesicular disease virus
Source: Evol Med Public Health. 2015 Oct 27;2015(1):289–303. doi: 10.1093/emph/eov026 (PMC4661520; doi:10.1093/emph/eov026)
Supplement: Supplementary Data [file supp_eov026_Bruhn_et_al_supplementary_tables_EMPH.pdf]

SUPPLEMENTARY TABLE S1

Global occurrence of swine vesicular disease 1966 - 2013

Compiled by N.J. Knowles, and last updated 22 November 2013.

Accessed at http://www.svd.org.uk/occurrence.htm on the 2. Feb. 2014.

Occurrence of Swine Vesicular Disease (1966 to 1980)

Country	1966	1970	1971	1972	1973	1974	1975	1976	1977	1978	1979	1980
**Austria**	-	-	-	3	>13	5	3	6	-	1	+ (a)	-
**Belgium**	-	-	-	-	1 (b)	-	-	-	-	-	6	-
**Bulgaria**	-	-	+	-	-	-	-	-	-	-	-	-
**China[g]**	-	-	-	-	-	-	-	-	-	-	-	-
**France**	-	-	-	-	+	-	3	-	-	-	-	-
**Germany (East)[g]**	-	-	-	-	-	-	-	-	-	-	-	-
**Germany (West)**	-	-	-	-	2	2	11	2	1	2	-	1
**Great Britain**	-	-	-	13	137	187	45	3	18	-	43	60
**Greece**	-	-	-	-	-	-	-	-	-	-	1	-
**Hong Kong**	-	1	10	2	2	4	3	3	1	-	1	2
**Italy**	2	-	-	>23	+	32	23	1	39	21	9	29
**Japan**	-	-	-	-	16	-	2 (c)	6 (d)	-	-	-	-
**Malta**	-	-	-	-	-	-	>100	-	-	-	-	-
**Netherlands**	-	-	-	-	-	-	1	-	-	-	-	-
**Poland**	-	-	-	2	+ (e)	-	-	-	-	-	-	-
**Portugal**	-	-	-	-	-	-	-	-	-	-	-	-
**Romania**	-	-	-	? (f)	+	-	-	-	-	-	-	-
**Spain**	-	-	-	-	-	-	-	-	-	-	-	-
**Switzerland**	-	-	-	-	1	-	-	-	-	-	-	-
**Taiwan Province of China**	-	-	-	-	-	-	-	-	-	-	-	-
**USSR**	-	-	-	+	-	-	+	-	-	-	-	-

Occurrence of Swine Vesicular Disease (1981 to 1992)

Country	1981	1982	1983	1984	1985	1986	1987	1988	1989	1990	1991	1992
**Austria**	-	-	-	-	-	-	-	-	-	-	-	-
**Belgium**	-	-	-	-	-	-	-	-	-	-	-	1[h]
**Bulgaria**	-	-	-	-	-	-	-	-	-	-	-	-
**China[g]**	-	-	-	-	-	-	-	-	-	-	-	-
**France**	-	1	3	-	-	-	-	-	-	-	-	-
**Germany (East)[g]**	-	-	-	-	-	-	-	-	-	-	-	-
**Germany (West)**	1	1	-	-	1 (b)	-	-	-	-	-	-	-
**Great Britain**	12	14	-	-	-	-	-	-	-	-	-	-
**Greece**	-	-	-	-	-	-	-	-	-	-	-	-
**Hong Kong**	5	-	-	1	3	-	6	2	2	-	4	-
**Italy**	5	8	4	1	-	-	-	1	1	-	6	31
**Japan**	-	-	-	-	-	-	-	-	-	-	-	-
**Malta**	-	-	-	-	-	-	-	-	-	-	-	-
**Netherlands**	-	-	-	-	-	-	-	-	-	-	-	6
**Poland**	-	-	-	-	-	-	-	-	-	-	-	-
**Portugal**	-	-	-	-	-	-	-	-	-	-	-	-
**Romania**	-	-	-	-	-	-	+	-	-	-	-	-
**Spain**	-	-	-	-	-	-	-	-	-	-	-	-
**Switzerland**	-	-	-	-	-	-	-	-	-	-	-	-
**Taiwan Province of China**	-	-	-	-	-	-	-	-	-	-	-	-
**USSR**	-	-	-	-	-	-	-	-	-	-	-	-

Occurrence of Swine Vesicular Disease (1993 to 2004)

Country	1993	1994	1995	1996	1997	1998	1999	2000	2001	2002	2003	2004
**Austria**	-	-	-	-	-	-	-	-	-	-	-	-
**Belgium**	1 (h)	-	-	-	-	-	-	-	-	-	-	-
**Bulgaria**	-	-	-	-	-	-	-	-	-	-	-	-
**China (g)**	-	-	-	-	-	-	-	-	-	-	-	-
**France**	-	-	-	-	-	-	-	-	-	-	-	-
**Germany (East)(g)**	-	-	-	-	-	-	-	-	-	-	-	-
**Germany (West)**	-	-	-	-	-	-	-	-	-	-	-	-
**Great Britain**	-	-	-	-	-	-	-	-	-	-	-	-
**Greece**	-	-	-	-	-	-	-	-	-	-	-	-
**Hong Kong**	-	-	-	-	-	-	-	-	-	-	-	-
**Italy**	11	30	16	5	16	25	15	5	10	171 (k)	23 (k)	125
**Japan**	-	-	-	-	-	-	-	-	-	-	-	-
**Malta**	-	-	-	-	-	-	-	-	-	-	-	-
**Netherlands**	-	3	-	-	-	-	-	-	-	-	-	-
**Poland**	-	-	-	-	-	-	-	-	-	-	-	-
**Portugal**	-	-	1 (i)	-	-	-	-	-	-	-	1	1
**Romania**	-	-	-	-	-	-	-	-	-	-	-	-
**Spain**	3	-	-	-	-	-	1 (j)	-	-	-	-	-
**Switzerland**	-	-	-	-	-	-	-	-	-	-	-	-
**Taiwan Province of China**	-	-	-	-	+	+	-	+	?	-	-	-
**Former USSR**	-	-	-	-	-	-	-	-	-	-	-	-

Occurrence of Swine Vesicular Disease (2005-2016)

Country	2005	2006	2007	2008	2009	2010	2011	2012	2013	2014	2015	2016
**Austria**	-	-	-	-	-	-	-	-	-			
**Belgium**	-	-	-	-	-	-	-	-	-			
**Bulgaria**	-	-	-	-	-	-	-	-	-			
**China**[g]	-	-	-	-	-	-	-	-	-			
**France**	-	-	-	-	-	-	-	-	-			
**Germany (East)**[g]	-	-	-	-	-	-	-	-	-			
**Germany (West)**	-	-	-	-	-	-	-	-	-			
**Great Britain**	-	-	-	-	-	-	-	-	-			
**Greece**	-	-	-	-	-	-	-	-	-			
**Hong Kong**	-	-	-	-	-	-	-	-	-			
**Italy**	13	51	89	65	18	4	25	7	1			
**Japan**	-	-	-	-	-	-	-	-	-			
**Malta**	-	-	-	-	-	-	-	-	-			
**Netherlands**	-	-	-	-	-	-	-	-	-			
**Poland**	-	-	-	-	-	-	-	-	-			
**Portugal**	-	-	1	-	-	-	-	-	-			
**Romania**	-	-	-	-	-	-	-	-	-			
**Spain**	-	-	-	-	-	-	-	-	-			
**Switzerland**	-	-	-	-	-	-	-	-	-			
**Taiwan Province of China**	-	-	-	-	-	-	-	-	-			
**Former USSR**	-	-	-	-	-	-	-	-	-			

a, unknown number of outbreaks-
b, not confirmed by laboratory investigations
c, one clinical outbreak and one farm where SVDV was isolated from healthy pigs
d, all from clinically normal pigs
e, 100-200 outbreaks ?
f, vesicular disease in pigs on a few farms, SVD suspected but no virus isolated (origin: pig meat from China)
g, dates and numbers of outbreaks not known
h, no clinical disease, diagnosis based on serology
i, virus isolated from one farm, however, approx. four more farms were sero-positive
j, sequence analysis suggested that the virus isolated was a laboratory contaminant
k, all were sub-clinical infections

SUPPLEMETARY TABLE S2

GARD analyses

GARD Analysis #	Genome Region	Alignment # seq/51 length	Analysis Converged	Breakpoint positions
1	Full	51/51 7412	NO	566, 3602***, 5206***
2	5'UTR-1B	51/51 1733	YES	566, 932***
3	1B-1D (external capsid)	51/51 2346	YES	1778***
4	1C-2D	51/51 2310	YES	2495*
5	1D-3C	51/51 2583	YES	3088***
6	2B-3C	51/51 2166	YES	4583***
7	3A-3D	51/51 2268	YES	-
8	3D-3'UTR	51/51 1496	YES	-
9	Full	26/51 7412	NO	566, 818, 1295**, 1727***, 3587***, 5216
10	Full	25/51 7412	YES	1295, 3184***
11	Full	23/51 7412	NO	566, 818, 1094***, 1658, 3689**
12	Full	15/51 7412	YES	572, 815, 1082***, 1883, 2271, 3839***, 5206
13	Full	51/51 7412	NO	566, 3482***, 5141***
14	5'UTR-3'UTR (trimmed)	15/51 7335	NO	574, 819, 1146***, 1878***, 3645, 3873, 5207, 5391
15	5'UTR (trimmed)-3'UTR (trimmed)	15/51 6938	YES	2702, 3529*, 3716***, 4946

Analysis #1 is the basic SVDV alignment (BSA). Analysis #10 has the complementary taxa to analysis #9. Analyses 11 and 12 are reduced sets of analysis 9. Analysis 13 only differs from #1 by trimmings in the 5'UTR and 3'UTR. No * means that there was no support from the Kishino-Hasegawa test, one, two and three stars mean support at p = 0.10, p = 0.05 and p = 0.01, respectively.

SUPPLEMENTARY TABLE S3

Historical occurrence of foot-and-mouth disease in 4 European countries

Reproduced from G. N.Mowat. Epidemiology of foot-and-mouth disease in Europe. *Rev. sci. tech. Off. Int. Epiz.*, 1986, **5** (2), 271-277.

	Belgium	France	Netherlands	United Kingdom
1957	328	99424	40	184
1958	588	14127	11	116
1959	57	6188	6	45
1960	180	7382	3	298
1961	121	2626	179	103
1962	6	199	5470	5
1963	67	28	2103	0
1964	2	56	146	0
1965	112	10	1426	1
1966	323	59	2194	34
1967	22	17	196	2210
1968	1	40	0	187
1969	3	35	0	0
1970	2	4	0	0
1971	1	8	21	0
1972	0	2	7	0
1973	0	1	0	0
1974	60	89	3	1
1975	21	2	2	0
1976	1	0	0	0
1977	0	0	1	0
1978	0	1	0	0
1979	0	21	0	0
1980	0	0	0	0
1981	0	18	0	2
1982	0	0	0	0
1983	0	0	4	0
1984	0	0	2	0
1985	0	0	0	0

SUPPLEMENTARY TABLE S4

Existing full or near-full genome sequenced SVDV isolates included in the basic SVDV alignment

Accession#	Isolate ref.	Origin	Date collected
KF963276	HKN/8/73	Tong Yan San Tsuen, Ping Shan, Y.L., Hong Kong	30. Mar. 1973
KF963277	HKN/18/74	Lo Shu Leng, Ta Kwu Ling, N.T., Hong Kong	11. Sep. 1974
KF963278	HKN/25/75	Shueng Tsuen, Pat Heung, Y.L., N.T., Hong Kong	1. Mar. 1975
KF963274	HKN/1/77	Pak Sah, Sap Pat Hueng, Y.L., Hong Kong	10. Mar. 1976
KF963275	HKN/5/77	Ping Che, Ta Kwu Ling, N.T., Hong Kong	7. May 1976
AY429470	HK'70	Hong Kong	
X54521	UKG/27/72	United Kingdom	11. Dec. 1972
D16364	J1'73	Japan	Nov.-Dec. 1973
D00435	H3'76	Japan	1976
AF268065	NET/1/92	Netherlands	1992
EU151450	ITL/1/92	Italy	1. Jun. 1992
EU151454	ITL/2/92	Italy	2. Sep. 1992
EU151451	ITL/6/92	Italy	16. Jun. 1992
EU151452	ITL/8/92	Italy	14. Aug. 1992
EU151448	ITL/18/92	Italy	5. Nov. 1992
EU151455	ITL/19/92	Italy	16. Nov. 1992
EU151456	ITL/1/97	Italy	30. Jan. 1997
EU151457	ITL/2/97	Italy	3. Feb. 1997
EU151449	ITL/3/97	Italy	7. Feb. 1997
EU151458	ITL/10/98	Italy	2. Nov. 1998
EU151459	ITL/1/99	Italy	11. Jan. 1999
EU151460	ITL/3/99	Italy	25. Jan. 1999
EU151461	ITL/5/99	Italy	4. Feb. 1999
EU151453	ITL/7/99	Italy	5. Feb. 1999

SUPPLEMENTARY TABLE S5

Analyses run in GARLI

Alignment	#Taxa	Length	Model
5'UTR	189	741	SYM [012345] eq. freq. + I + 4G
1A (VP4)	180	207	TIM2 [010232] + I + 4G
1B (VP2)	124	767	TIM2 [010232] + I + 4G
1C (VP3)	98	714	TIM2 [010232] eq. freq. + 4G
1D (VP1)	427	850	GTR + 4G
2A	197	450	TIM2 [010232] eq. freq. + I + 4G
2C	307	987	GTR + I + 4G
3C (Protease)	218	549	GTR + I + 4G
3D (Polymerase)	489	1386	GTR + I + 4G

SUPPLEMETARY TABLE S6

Parameter priors used in BEAST analyses

**Parameter**	**Prior**	**Bound**
CP1+2.kappa	LogNormal [1, 1.25], initial = 2	[0, inf.]
CP3.kappa	LogNormal [1, 1.25], initial = 2	[0, inf.]
CP1+2.mu	LogNormal [0, 1], initial = 1	[0, inf.]
CP3.mu	LogNormal [0, 1], initial = 1	[0, inf.]
frequencies	Uniform [0, 1], initial = 0.25	[0, 1]
CP1+2.alpha	Exponential [0.5], initial = 0.5	[0, inf.]
CP3.alpha	Exponential [0.5], initial = 0.5	[0, inf.]
clock.rate	Gamma [0.001, 1000], initial = 1	[0, inf.]
uced.mean	Gamma [0.001, 1000], initial = 1	[0, inf.]
ucld.mean	Gamma [0.001, 1000], initial = 1	[0, inf.]
ucld.stdev	Exponential [0.333333], initial = 0.3333...	[0, inf.]
treeModel.rootHeight	Using Tree Prior in [40.167123, inf.]	←
constant.popSize	Gamma [0.001, 1000], initial = 10	[0, inf.]
exponential.popSize	Gamma [0.001, 1000], initial = 10	[0, inf.]
logistic.popSize	Gamma [0.001, 1000], initial = 10	[0, inf.]
skyline.popSize	Exponential [1000], initial = 100	[0, inf.]
exponential.growthRate	Laplace [0, 0.023026], initial = 0.4	[-inf., inf.]
logistic.growthRate	Laplace [0, 0.017269], initial = 1	[-inf., inf.]
logistic.t50	Gamma [0.001, 1000], initial = 1	[0, inf.]
meanRate	Indirectly Specified Through Other Parameters	n/a
covariance	Indirectly Specified Through Other Parameters	n/a
coefficientOfVariation	Indirectly Specified Through Other Parameters	n/a
origin	Exponential [100], initial = 440	[0, inf.]
R0	LogNormal [1, 1], initial = 5	[0, inf.]
recoveryRate	Gamma [1, 1], initial = 0.05	[0, inf.]
samplingProbability	Beta [1, 1], initial = 0.05	[0, 1]
