# Supplementary material for: Viral meningitis epidemics and a single, recent, recombinant and anthroponotic origin of swine vesicular disease virus
Source: Evol Med Public Health. 2015 Oct 27;2015(1):289–303. doi: 10.1093/emph/eov026 (PMC4661520; doi:10.1093/emph/eov026)

SUPPLEMENTARY FIGURE S1

Results from the GARD recombination analysis

On the y-axis the numbers for the individual GARD analyses, which can be seen in Supplementary Table 2. Along the x-axis, genome positions and layout of an SVDV genome (H/3 76 D00435, Japan 1976). All recombination signals for all analyses are shown. Grey indicates no significant support from the Kishino-Hasegawa test in GARD. Yellow, orange, and red, indicate support at p = 0.1, p = 0.05 and p = 0.01 respectively (see Supplementary Table 2).

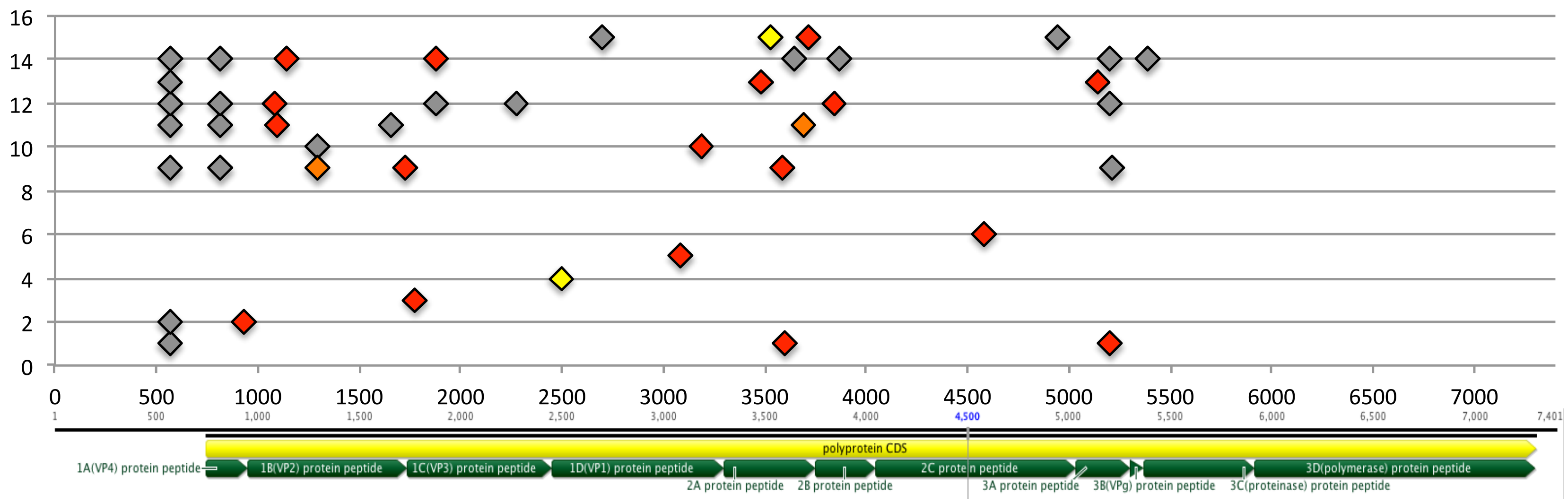

16
14
12
10
8
6
4
2
0
0
500
1000
1500
2000
2500
3000
3500
4000
4500
5000
5500
6000
6500
7000
1
500
1,000
1,500
2,000
2,500
3,000
3,500
4,000
4,500
5,000
5,500
6,000
6,500
7,000
7,401
polyprotein CDS
1A(VP4) protein peptide
1B(VP2) protein peptide
1C(VP3) protein peptide
1D(VP1) protein peptide
2A protein peptide
2B protein peptide
2C protein peptide
3A protein peptide
3B(VPg) protein peptide
3C(proteinase) protein peptide
3D(polymerase) protein peptide

SUPPLEMENTARY FIGURE S2

Trees from the maximum likelihood phylogenetic analysis

Results from the phylogenetic analyses of 8 of the 9 chosen genomic sections (see Fig. 1 for 3D section) ordered according to their genomic positions. All show an unrooted tree with no taxa designations, except that branches leading to an SVDV isolate are coloured pink. The branch leading to the root of the SVDV cluster is shown in black, like the remainder of the tree. When in some analyses part of the branch leading to the SVDV cluster is pink, this is because there is an SVDV sequence (i.e. ITL/1/66) at the root of the cluster at the beginning of the colour change. The unrooted trees give an immediate overview of the relation between SVDV and all other sequences in the analysis, including relative (and if conferring with the 0.1 substitution per site bar, also absolute) distance to the nearest neighbours. A single analysis, that of 1A (VP4) does not show SVDV as monophyletic – it is clear, from the unrooted tree that this analysis suffers from some very long branches. Even though the analysis only contains 180 taxa in total (see Supplementary Table 5), it is clear that more sequences could have been left out. Furthermore, the only closely related sequences match with what is found for the other 3 capsid protein coding sections. All sections, except 5′ UTR, are also illustrated with taxa designations and support values (aBayes) for the closest related sequences to the SVDV cluster. These are cut-outs of mid-point rooted versions of the trees. The 5’UTR is not illustrated in this manner, since the structure and taxa of the tree renders such a transformation uninformative (see main text).

5’UTR

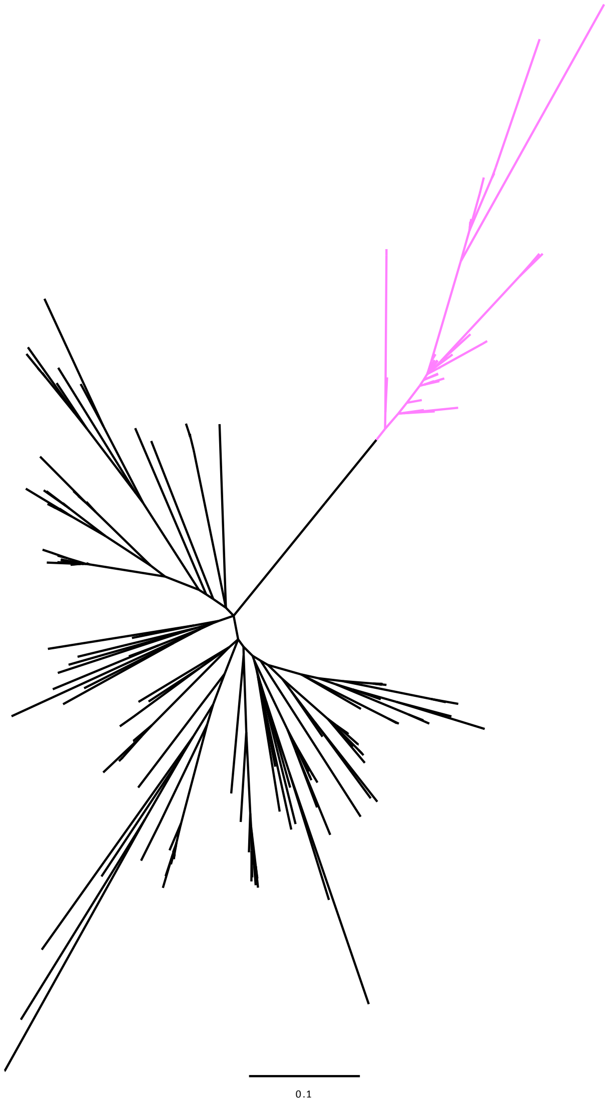

1A (VP4)

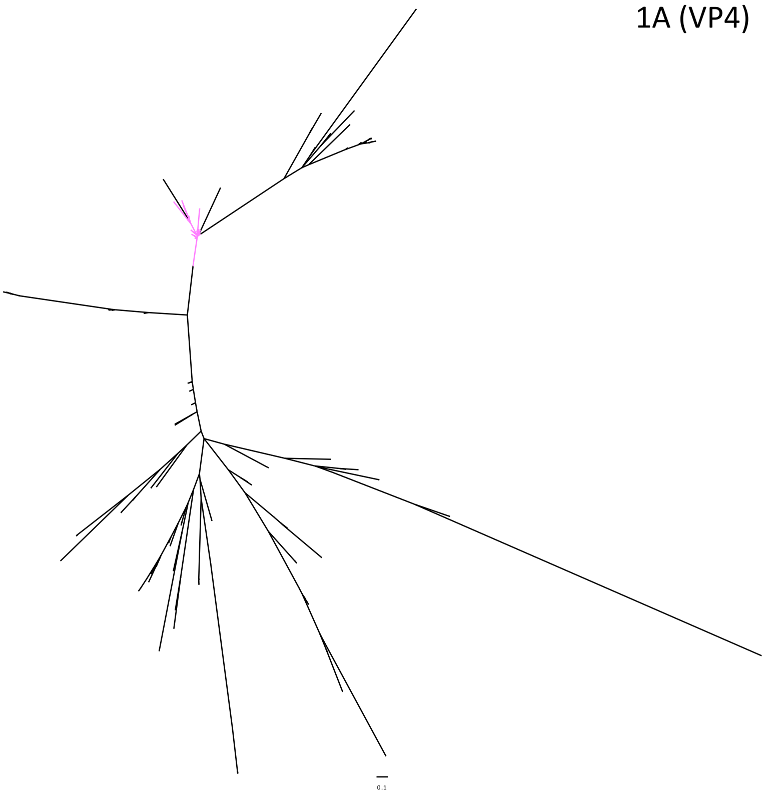
0.1

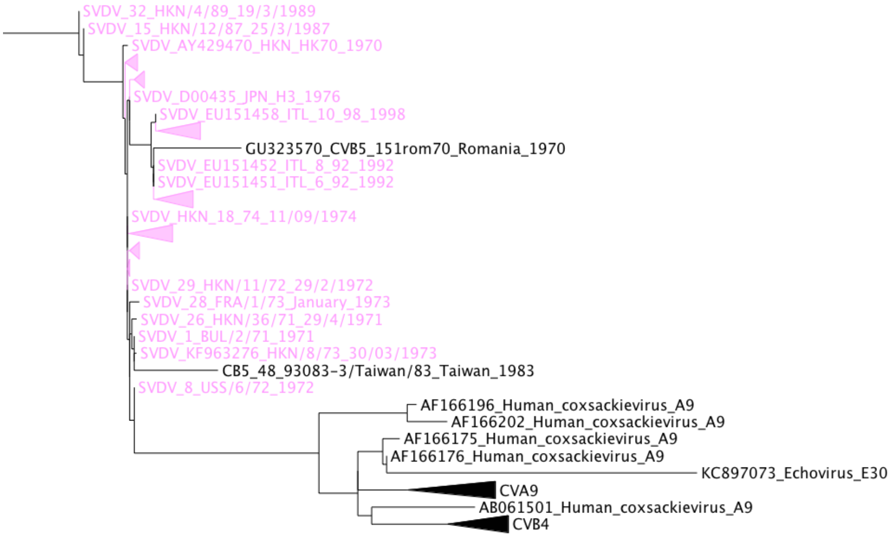
SVDV_32_HKN/4/89_19/3/1989
SVDV_15_HKN/12/87_25/3/1987
SVDV_AY429470_HKN_HK70_1970
SVDV_D00435_JPN_H3_1976
SVDV_EU151458_ITL_10_98_1998
GU323570_CVB5_151rom70_Romania_1970
SVDV_EU151452_ITL_8_92_1992
SVDV_EU151451_ITL_6_92_1992
SVDV_HKN_18_74_11/09/1974
SVDV_29_HKN/11/72_29/2/1972
SVDV_28_FRA/1/73_January_1973
SVDV_26_HKN/36/71_29/4/1971
SVDV_1_BUL/2/71_1971
SVDV_KF963276_HKN/8/73_30/03/1973
CB5_48_93083-3/Taiwan/83_Taiwan_1983
SVDV_8_USS/6/72_1972
AF166196_Human_coxsackievirus_A9
AF166202_Human_coxsackievirus_A9
AF166175_Human_coxsackievirus_A9
AF166176_Human_coxsackievirus_A9
KC897073_Echovirus_E30
CVA9
AB061501_Human_coxsackievirus_A9
CVB4

1B (VP2)

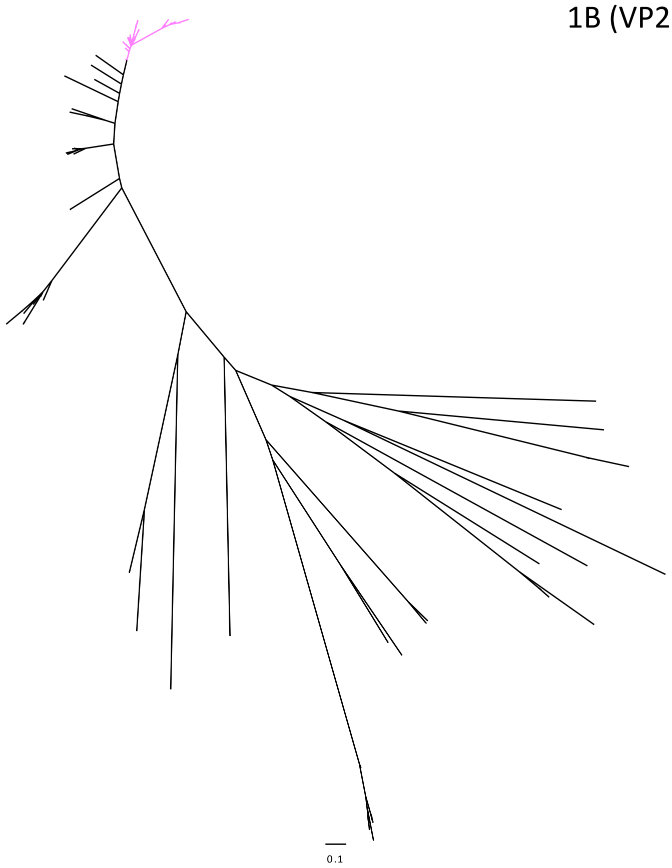
0.1

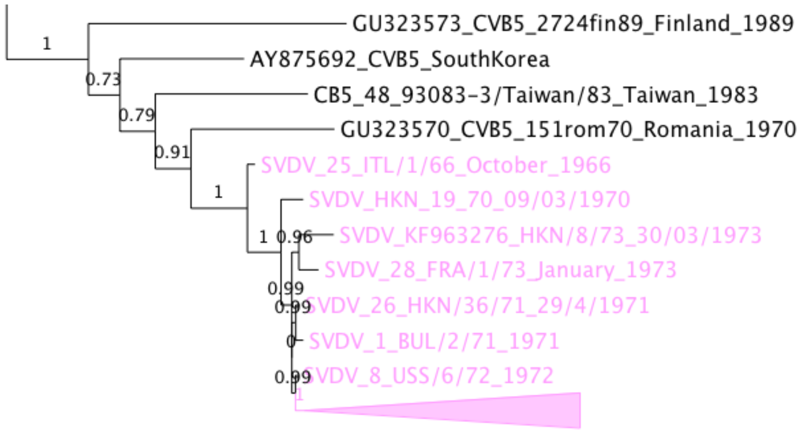
GU323573_CVB5_2724fin89_Finland_1989
AY875692_CVB5_SouthKorea
CB5_48_93083-3/Taiwan/83_Taiwan_1983
GU323570_CVB5_151rom70_Romania_1970
SVDV_25_ITL/1/66_October_1966
SVDV_HKN_19_70_09/03/1970
SVDV_KF963276_HKN/8/73_30/03/1973
SVDV_28_FRA/1/73_January_1973
SVDV_26_HKN/36/71_29/4/1971
SVDV_1_BUL/2/71_1971
SVDV_8_USS/6/72_1972
1
0.73
0.79
0.91
1
1
0.96
0.99
0.99
0
0.99
1

1C (VP3)

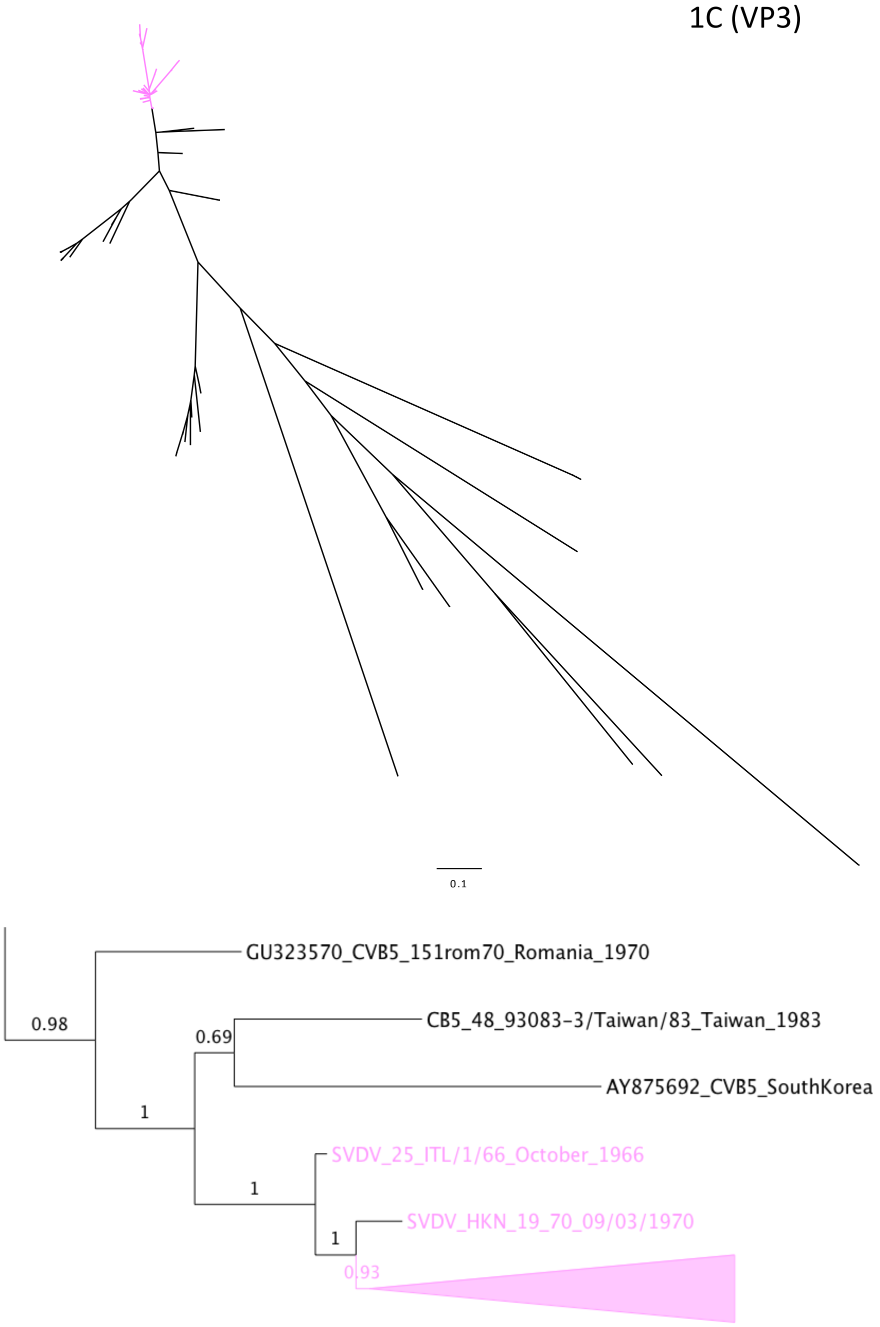
0.1
GU323570_CVB5_151rom70_Romania_1970
CB5_48_93083-3/Taiwan/83_Taiwan_1983
AY875692_CVB5_SouthKorea
SVDV_25_ITL/1/66_October_1966
SVDV_HKN_19_70_09/03/1970
0.98
0.69
1
1
1
0.93

1D (VP1)

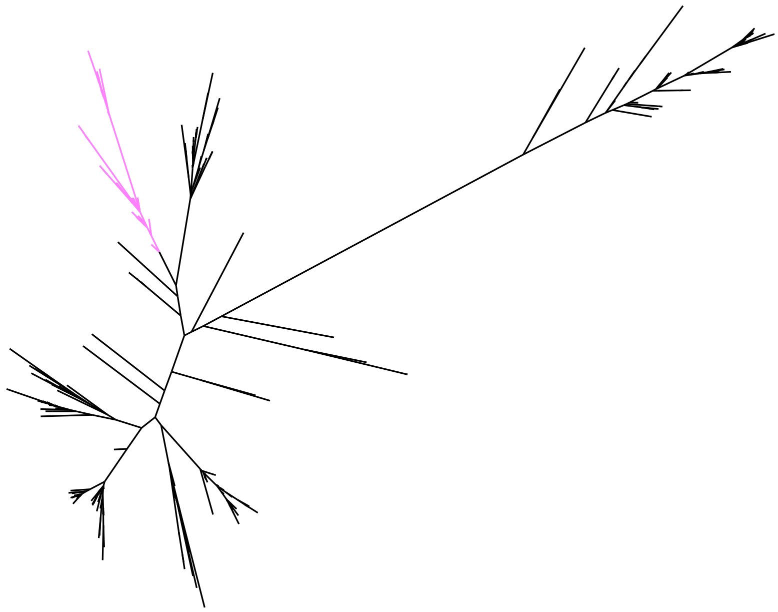

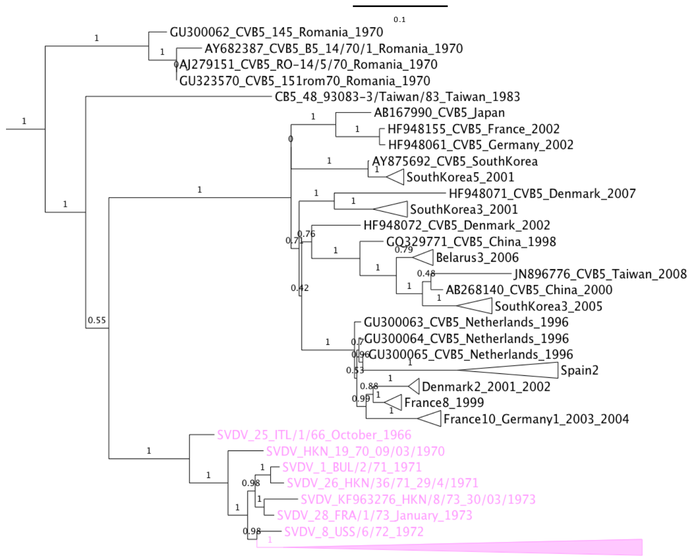
0.1
GU300062_CVB5_145_Romania_1970
AY682387_CVB5_B5_14/70/1_Romania_1970
AJ279151_CVB5_RO-14/5/70_Romania_1970
GU323570_CVB5_151rom70_Romania_1970
CB5_48_93083-3/Taiwan/83_Taiwan_1983
AB167990_CVB5_Japan
HF948155_CVB5_France_2002
HF948061_CVB5_Germany_2002
AY875692_CVB5_SouthKorea
SouthKorea5_2001
HF948071_CVB5_Denmark_2007
SouthKorea3_2001
HF948072_CVB5_Denmark_2002
GQ329771_CVB5_China_1998
Belarus3_2006
JN896776_CVB5_Taiwan_2008
AB268140_CVB5_China_2000
SouthKorea3_2005
GU300063_CVB5_Netherlands_1996
GU300064_CVB5_Netherlands_1996
GU300065_CVB5_Netherlands_1996
Spain2
Denmark2_2001_2002
France8_1999
France10_Germany1_2003_2004
SVDV_25_ITL/1/66_October_1966
SVDV_HKN_19_70_09/03/1970
SVDV_1_BUL/2/71_1971
SVDV_26_HKN/36/71_29/4/1971
SVDV_KF963276_HKN/8/73_30/03/1973
SVDV_28_FRA/1/73_January_1973
SVDV_8_USS/6/72_1972

2A

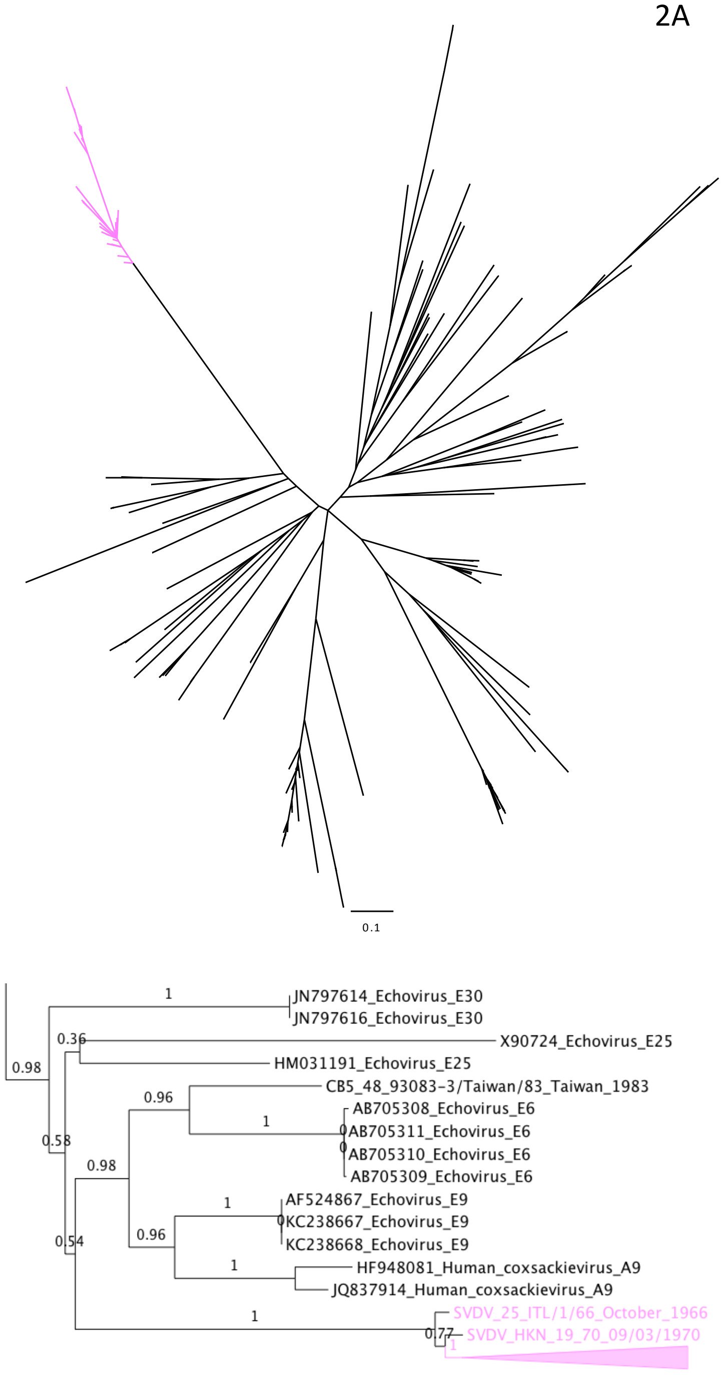
0.1
1
0.98
0.36
0.58
0.96
0.98
0.54
0.96
0.77
JN797614_Echovirus_E30
JN797616_Echovirus_E30
X90724_Echovirus_E25
HM031191_Echovirus_E25
CB5_48_93083-3/Taiwan/83_Taiwan_1983
AB705308_Echovirus_E6
AB705311_Echovirus_E6
AB705310_Echovirus_E6
AB705309_Echovirus_E6
AF524867_Echovirus_E9
KC238667_Echovirus_E9
KC238668_Echovirus_E9
HF948081_Human_coxsackievirus_A9
JQ837914_Human_coxsackievirus_A9
SVDV_25_ITL/1/66_October_1966
SVDV_HKN_19_70_09/03/1970

2C

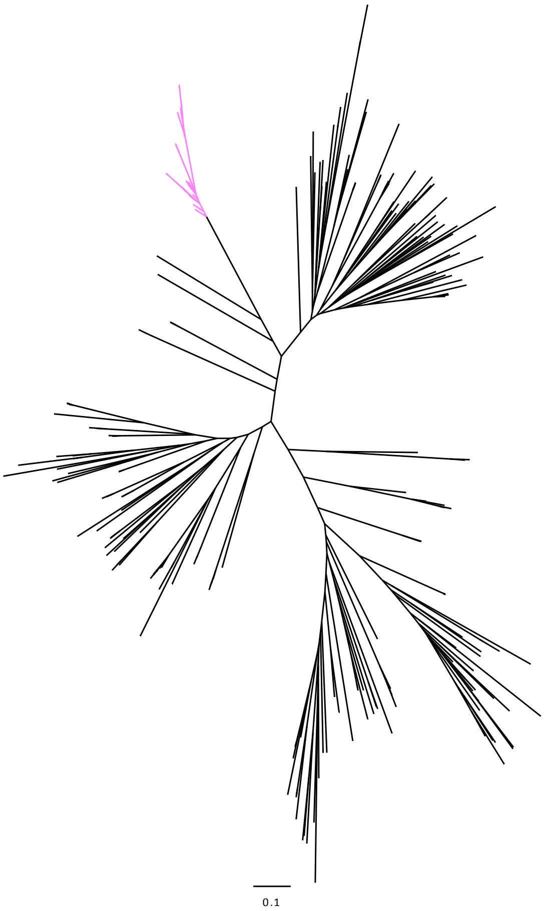

0.1

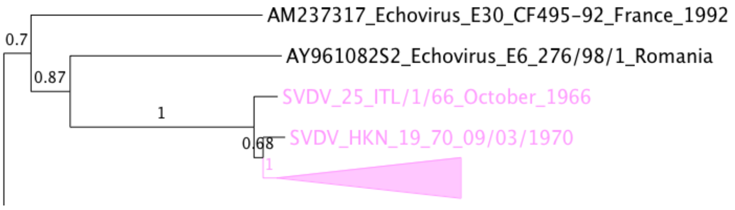

AM237317_Echovirus_E30_CF495-92_France_1992
AY961082S2_Echovirus_E6_276/98/1_Romania
SVDV_25_ITL/1/66_October_1966
SVDV_HKN_19_70_09/03/1970
0.7
0.87
1
0.68
1

3C

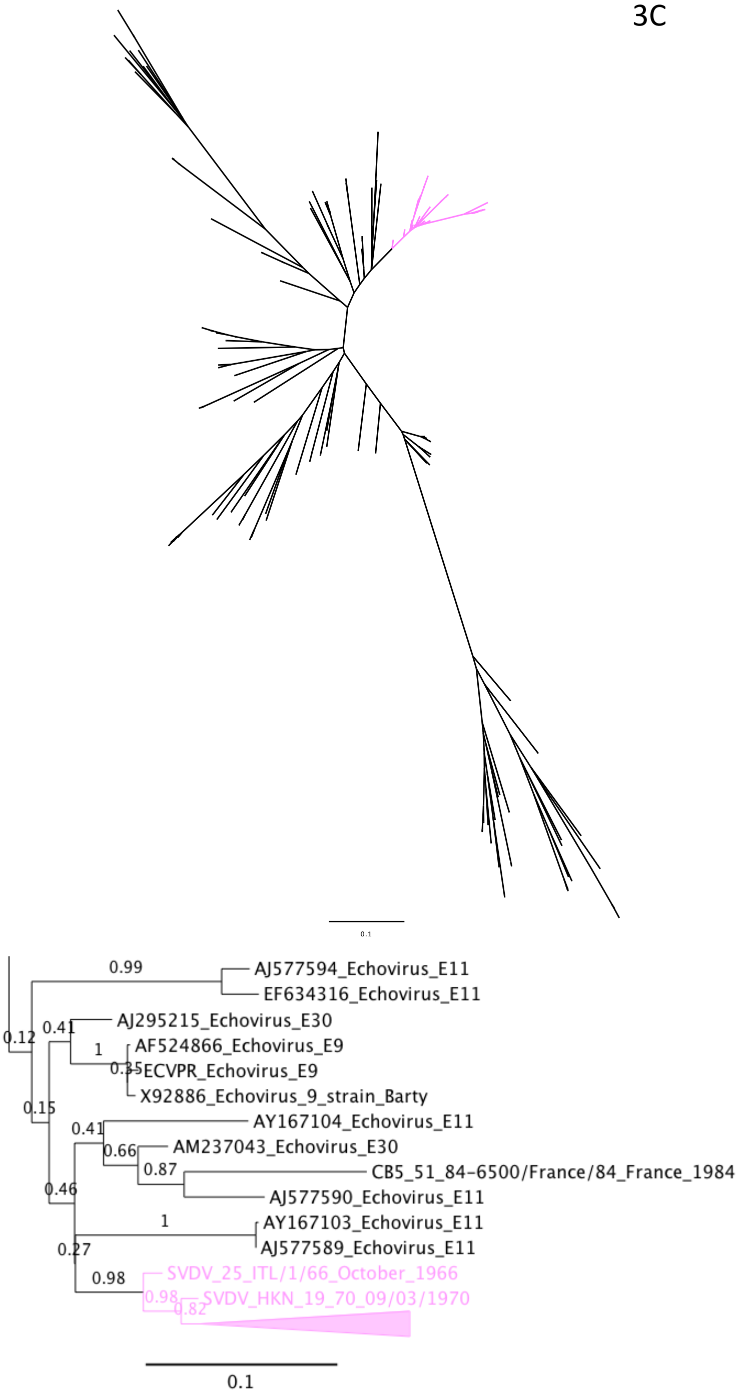
0.1
0.99
AJ577594_Echovirus_E11
EF634316_Echovirus_E11
0.12
0.41
AJ295215_Echovirus_E30
1
AF524866_Echovirus_E9
0.35
ECVPR_Echovirus_E9
X92886_Echovirus_9_strain_Barty
0.15
0.41
AY167104_Echovirus_E11
0.66
AM237043_Echovirus_E30
0.87
CB5_51_84-6500/France/84_France_1984
0.46
AJ577590_Echovirus_E11
1
AY167103_Echovirus_E11
0.27
AJ577589_Echovirus_E11
0.98
SVDV_25_ITL/1/66_October_1966
0.98
SVDV_HKN_19_70_09/03/1970
0.82
0.1

SUPPLEMENTARY FIGURE S3

Dating estimate trees from the BEAST analyses

Results annotated onto the maximum clade credibility tree for each of the four analyses marked in bold in Table 1. Blue bars indicate 95% HPD intervals for the age of the given node, and numbers indicate posterior support for the clusters (see also Methodology).

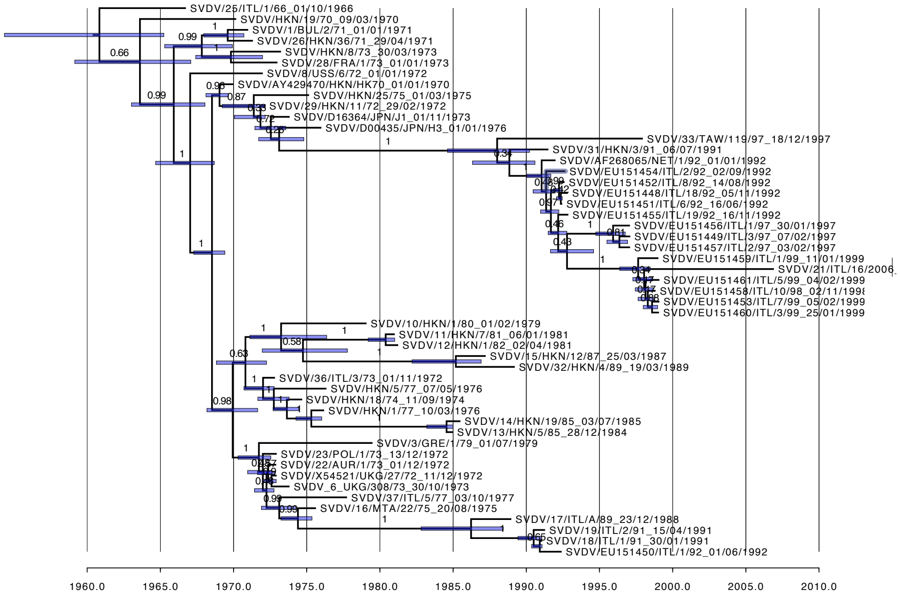

1C-1D (VP3-VP1), 400 codon section, Coalescent Bayesian Skyline

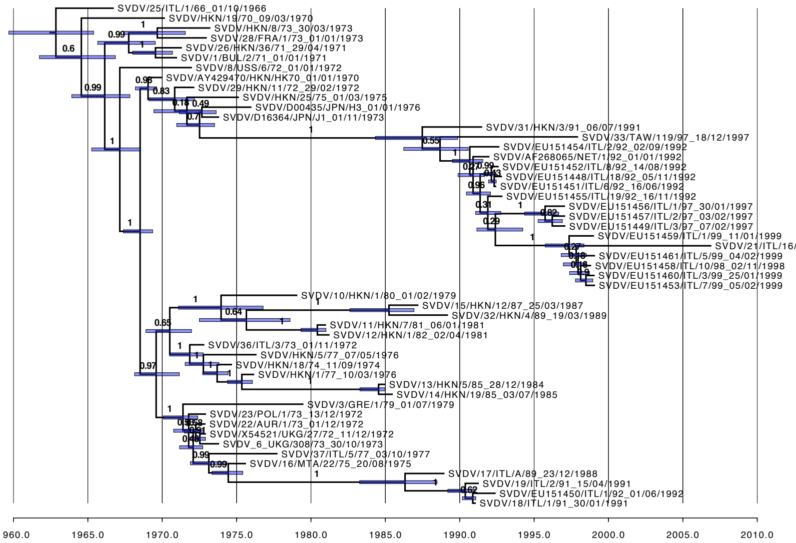

1C-1D (VP3-VP1), 400 codon section, Epidemiology Birth-Death Model

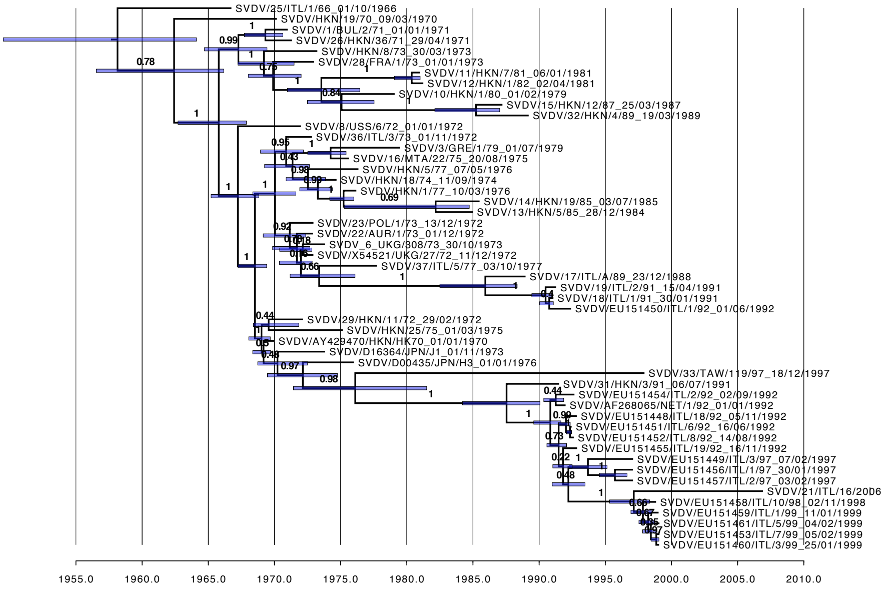

3C-3D (Protease-Polymerase), full region, Coalescent Bayesian Skyline

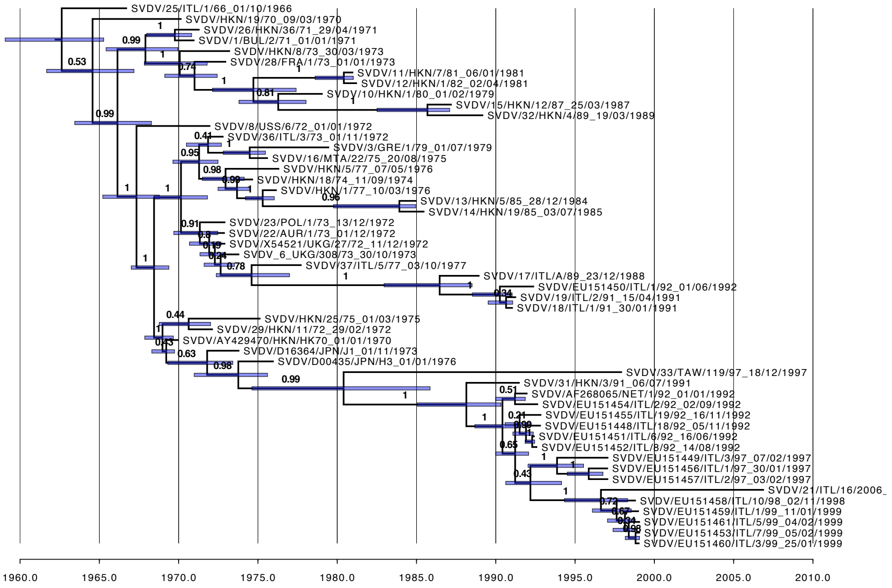

3C-3D (Protease-Polymerase), full regionEpidemiology Birth-Death Model

SUPPLEMENTARY FIGURE S4

Marginal density plots for the tree model root height parameter

Marginal density plots for the tree model roots height (age of most recent common ancestor) for the 4 best scoring analysis (shown in bold in Table 1) in the BEAST dating estimates. On top, results from the Bayesian coalescent skyline prior based analyses for each of the two analysed segments, the capsid section in grey and the protease-polymerase section in blue. Below, results from the Epidemiological birth-death prior. The results correspond to the root of the trees shown in Supplementary Fig. S3. See also Methodology and Table 1 for further details.

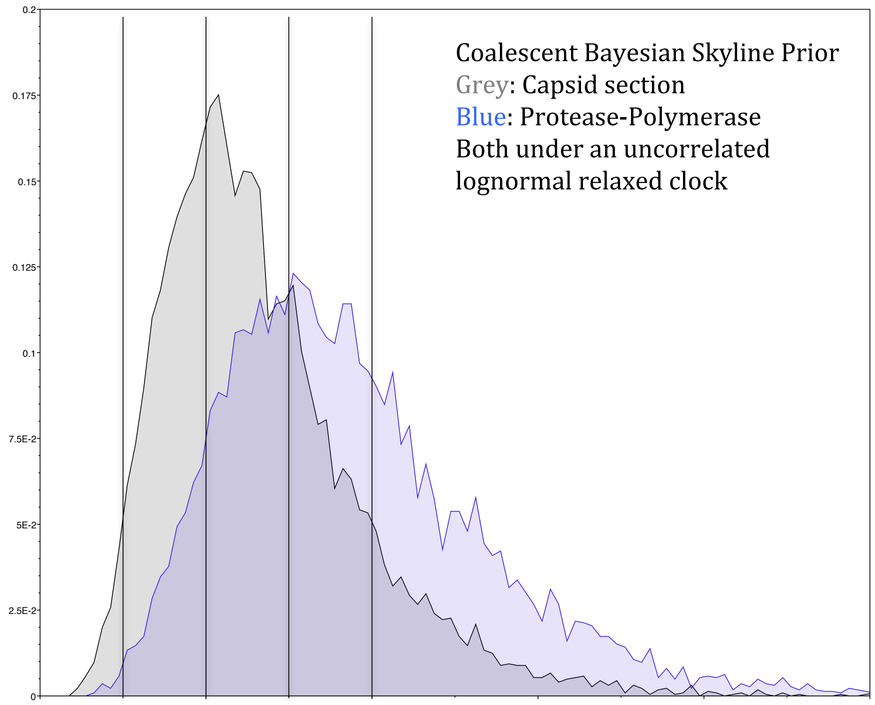

1. Jun. 1964   1. Jun. 1959

1. Dec. 1961   1. Dec. 1956

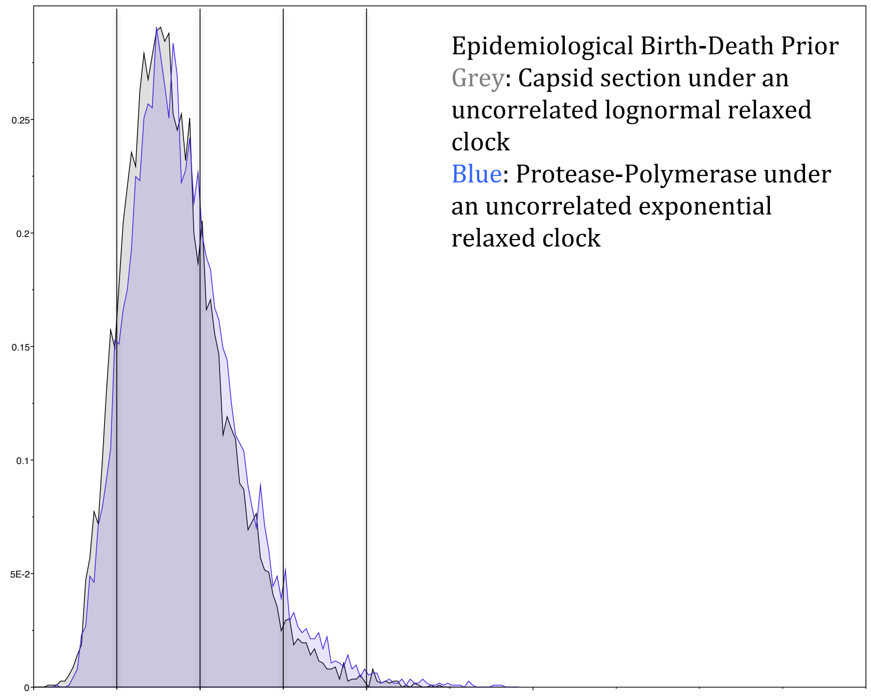

Supplement: Supplementary Data [file supp_eov026_Bruhn_et_al_SVDV_supplementary_figures_EMPH.pdf]
